# CRISPR/Cas9‐mediated mutation of *Eil1* transcription factor genes affects exogenous ethylene tolerance and early flower senescence in *Campanula portenschlagiana*

**DOI:** 10.1111/pbi.14200

**Published:** 2023-10-12

**Authors:** Inger B. Holme, Christina R. Ingvardsen, Giuseppe Dionisio, Dagmara Podzimska‐Sroka, Kell Kristiansen, Anders Feilberg, Henrik Brinch‐Pedersen

**Affiliations:** ^1^ Department of Agroecology, Faculty of Technical Sciences Aarhus University Slagelse Denmark; ^2^ PKM Innovation ApS Odense Denmark; ^3^ Department of Biological and Chemical Engineering, Faculty of Technical Sciences Aarhus University Aarhus Denmark

**Keywords:** Ethylene tolerance, flower senescence, CRISPR/Cas9, *Eil1* transcription factors, *Campanula portenschlagiana*

## Abstract

Improving tolerance to ethylene‐induced early senescence of flowers and fruits is of major economic importance for the ornamental and food industry. Genetic modifications of genes in the ethylene‐signalling pathway have frequently resulted in increased tolerance but often with unwanted side effects. Here, we used CRISPR/Cas9 to knockout the function of two *CpEil1* genes expressed in flowers of the diploid ornamental plant *Campanula portenschlagiana*. The ethylene tolerance in flowers of the primary mutants with knockout of only one or all four alleles clearly showed increased tolerance to exogenous ethylene, although lower tolerance was obtained with one compared to four mutated alleles. The allele dosage effect was confirmed in progenies where flowers of plants with zero, one, two, three and four mutated alleles showed increasing ethylene tolerance. Mutation of the *Cpeil1* alleles had no significant effect on flower longevity and endogenous flower ethylene level, indicating that *CpEil1* is not involved in age‐dependent senescence of flowers. The study suggests focus on *EIN3/Eils* expressed in the organs subjected to early senescence for obtaining tolerance towards exogenous ethylene. Furthermore, the observed allelic dosage effect constitutes a key handle for a gradual regulation of sensitivity towards exogenous ethylene, simultaneously monitoring possibly unwanted side effects.

## Introduction

The gaseous hormone ethylene produced in plants during development plays a key role in growth, senescence of leaves and flowers and fruit ripening (Iqbal *et al*., 2017). Moreover, endogenous ethylene is produced in response to biotic and abiotic stresses, inducing various stress defence reactions (Ku *et al*., 2018).

Exogenous ethylene exposures encountered during transportation and storage also have impacts on plants and can be harmful depending on the species and the developmental stage. Non‐climacteric flowers, such as Compositae and Umbelliferae, show very low sensitivity to exogenous ethylene, whereas families with climacteric flowers like Campanulaceae and Rosaceae are very sensitive with ethylene inducing early senescence, that is, petal senescence in non‐senescent flowers (Woltering and Van Doorn, 1988). Similarly, climacteric fruits are highly sensitive to ethylene (Paul *et al*., 2012). Genetic handles for modulating exogenous ethylene tolerance in commercial plants with climacteric flowers and fruits are most wanted.

**Figure 1:**
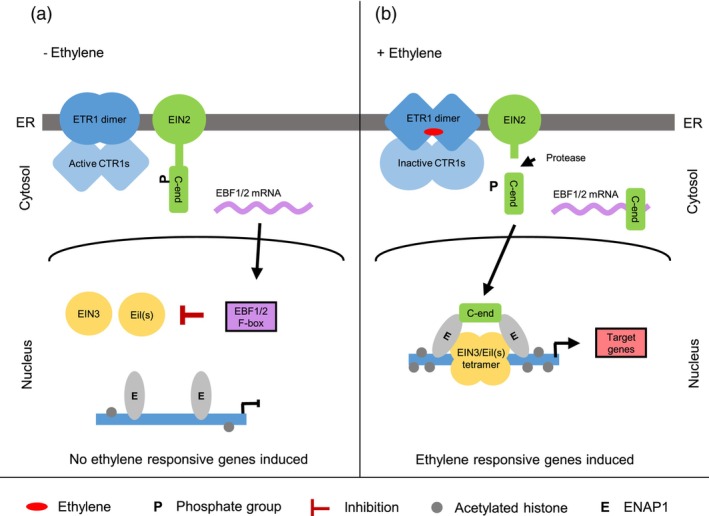
The key components of the ethylene‐signalling pathway. (a) Ethylene is perceived by a family of receptors in the ER membrane, represented by ETR1 (Shakeel *et al*., 2013). In the absence of ethylene, the ethylene receptors activate the serine–threonine protein kinase CTR1. CTR1 phosphorylates the C‐terminal end (C‐end) of EIN2, repressing the cleavage of the C‐terminal end (Ju *et al*., 2012). Two F‐box proteins, EBF1 and EBF2, are translocated to the nucleus, where they mediate degradation of the EIN3/Eil(s) transcription factors (Li *et al*., 2015). The EIN2 nuclear‐associated protein 1 (ENAP1) interacts with the histones (Zhang *et al*., 2017). The level of histone acetylation is low. (b) Ethylene binding to the ETR1 dimer induces a conformational change in the receptor, resulting in inactivation of CTR1. EIN2 is less phosphorylated, and the end is cleaved off by an unknown mechanism. In the cytosol, the EIN2 C‐end binds to the 3′ untranslated region of EBF1 and EBF2 mRNA, repressing the translation (Li *et al*., 2015). In the nucleus, the EIN2 C‐end interacts with ENAP1, resulting in an elevated level of histone acetylation and the binding of EIN3/Eil(s) tetramers to the promoter regions of target genes (Wu *et al*., 2020; Zhang *et al*., 2017).

Ethylene is perceived by a family of receptors localized in the endoplasmic reticulum (ER) membrane (Shakeel *et al*., 2013; Figure 1). There are five ethylene receptor genes (*ETR1*, *ERS1*, *ETR2*, *EIN4* and *ERS2*). Ethylene binding induces a conformational change in the receptors, resulting in inactivation of the Constitutive Triple Response 1 (CTR1; Figure 1). Ethylene‐insensitive 2, constitutive triple response 1 and EIN2 nuclear‐associated protein 1 (Ju *et al*., 2012; Qiao *et al*., 2012; Wen *et al*., 2012). The C‐end of EIN2 (EIN2‐C) has a dual role (Figure 1). In the cytosol, EIN2‐C binds to the 3′ untranslated region of EBF1 and EBF2 mRNA, repressing their translation and thereby preventing the degradation of the EIN3/Eils transcription factors (TFs) (Li *et al*., 2015). In the nucleus, EIN2‐C interacts with EIN2 Nuclear‐Associated Protein1 (ENAP1), resulting in an elevated level of histone acetylation. This allows the binding of ethylene‐stabilized EIN3/Eil homo‐ or heterotetramers to the promoter regions of EIN3/Eil‐targeted genes, thereby activating a cascade of genes involved in the downstream ethylene responses (Liu *et al*., 2016; Solano *et al*., 1998; Wu *et al*., 2020; Zhang *et al*., 2016, 2017).

Several strategies have been devised to control the exogenous ethylene response in climacteric flowers and fruits. One strategy is chemical blockage of ethylene perception. Chemical treatments of plants are, however, associated with concerns for human health and/or the environment. Genetic modification is another way of modulating ethylene sensitivity. Different mutations in *ETR1* have been discovered in *Arabidopsis*, including *etr1‐1* conferring dominant ethylene insensitivity (Chang *et al*., 1993). Insertion of *etr1‐1* under the control of a constitutive promoter reduced sensitivity to exogenous ethylene in petunia (Wilkinson *et al*., 1997). Results from petunia suggest that constitutive expression gives unwanted side effects (Clark *et al*., 1999). Tissue‐specific and spatiotemporal expressed promoters have been used with more success (Gehl *et al*., 2018; Ghayoor Karimiani *et al*., 2015; Sanikhani *et al*., 2008; Sriskandarajah *et al*., 2007; Winkelmann *et al*., 2016). *EIN2*, acting as a positive ethylene regulator in the ethylene‐signalling pathway, is an obvious candidate for down‐regulation/silencing via genetic modifications. *EIN2* has been the target of modification, using CRISPR/Cas9 in *Parasponia andersonii* and antisense in petunia (Shibuya *et al*., 2004; Van Zeijl *et al*., 2018). In all cases, plants had a reduced sensitivity towards exogenously applied ethylene. However, although the petunia flowers showed increased flower longevity after ethylene treatment, the plants also showed changes in root morphology and premature death.

Specific *EIN3/Eils* genes involved in exogenously ethylene‐induced early senescence are also strong candidates for down‐regulation or silencing. EIN3 and Eil proteins belong to a small family of TFs which are considered the key transcriptional regulators of the ethylene response. There are six genes encoding EIN3/Eil members in *Arabidopsis* (Chao *et al*., 1997; Guo and Ecker, 2004), four in tomato (Tieman *et al*., 2001; Yokotani *et al*., 2003), five in tobacco (Kosugi and Ohashi, 2000; Rieu *et al*., 2003), 10 in pear, four in peach, five in mei and five in strawberry (Cao *et al*., 2017). The proteins are highly conserved within and between species. Especially the N‐termini containing the DNA‐binding domain (DBD) are well conserved. The N‐termini include a proline‐rich region and five small, scattered clusters of basic amino acids (Basic domains; BD I–BD V) (Chao *et al*., 1997). The DBD is composed of six α‐helices and five short helical turns (η1–η5). This region also includes BD III, BD IV and the proline‐rich region, shown to be necessary for DNA binding in EIN3 (Song *et al*., 2015).

Ethylene‐stabilized EIN3/Eils activate a transcriptional cascade of genes involved in developmental processes and stress responses, either directly or through activation of other TFs. The mechanisms behind the fine‐tuning of the EIN3/Eil‐mediated gene activations in time and space and under different environmental conditions are, however, still rather unclear. In *Arabidopsis*, it is known that tuning of EIN3‐ and Eil1‐mediated gene activation can be modulated by epigenetic regulation, EIN3/Eil1 protein stability regulation, suppression of transcriptional activity (through crosstalk with jasmonic acid and gibberellins) or by collaboration between EIN3/Eil1 and other TFs to modulate the expression of a gene (Dolgikh *et al*., 2019; Ju and Chang, 2015).

The different EIN3/Eils found within a species might have different functions in different tissues. Expression profiles of the various *EIN3/Eil* genes within a species show variation in the expression levels in different tissues, indicating their variable role in specific tissues (Cao *et al*., 2017; Jyoti *et al*., 2021; Li *et al*., 2019a). Moreover, biotic or abiotic stresses further variate the expression level of *EIN3/Eils* (He *et al*., 2020; Li *et al*., 2019b). Analysis of *cis*‐acting elements within promoters of different *EIN3/Eils* shows growth‐ and development‐related, phytohormone and stress responsive elements which might be responsible for their spatiotemporal expression levels (He *et al*., 2020; Jyoti *et al*., 2021; Li *et al*., 2019a, b; Salih *et al*., 2020).

Specific EIN3/Eils seem to have special roles for flower senescence. Studies in carnation, peony, petunia and rose have shown that up‐regulation of expression levels of specific *EIN3/Eil* gene(s) in the flowers is correlated with sensitivity to exogenous ethylene (Iordachescu and Verlinden, 2005; Liu *et al*., 2016; Singh *et al*., 2019; Wang *et al*., 2013). In *Campanula medium*, a naturally induced 7‐bp deletion in the ORF of *Eil2*, isolated from flowers, correlated with flowers insensitive to exogenously applied ethylene (Jensen *et al*., 2016). The potential of down‐regulating expression of specific *EIN3/Eil* genes to obtain ethylene insensitive flowers by using genetic modifications is further strengthened by a paper showing that VIGS‐mediated silencing in two petunia *Eils*, *PhEil1* and *PhEil2*, delayed flower senescence (Liu *et al*., 2016).

*Campanula portenschlagiana* is a diploid perennial plant widely used in the ornamental industry. The climacteric flowers of *C. portenschlagiana* are very sensitive to exogenous ethylene. Here, two genes in *C. portenschlagiana*, *CpEil1a* and *CpEil1b*, both expressed in flowers, were targeted in their DNA‐binding domains by CRISPR/Cas9. The results demonstrate that the response to exogenous ethylene is regulated in a *CpEil1* allele dosage specific manner and points towards *EIN3/Eils* as key handles for gradual modulation of ethylene sensitivity.

## Results

### Cloning and sequencing of *C. portenschlagiana Eil1a* and *Eil1b*

Partial CDS for *Eil1a* (*CpEil1a*_allele1, OM925995; *CpEil1a*_allele2, OM925996; both 2262 bp) and *Eil1b* (*CpEil1b*_allele1, OM925997; *CpEil1b*_allele2, OM925998; both 924 bp) from *C. portenschlagiana* ‘PKMp11’ were cloned. This added 1668 and 333 bp, respectively, to the previously published *Eil1a* (KX058425.1; 594 bp) and *Eil1b* (KX058426.1; 591 bp) gene fragments (Jensen *et al*., 2016). Single nucleotide polymorphisms (SNPs) found between the two alleles in each *CpEil1* gene enabled us to assign the CRISPR/Cas9‐induced mutations to specific alleles (Figure S1).

### Primary mutants obtained with the CRISPR/Cas9 construct

**Figure 2:**
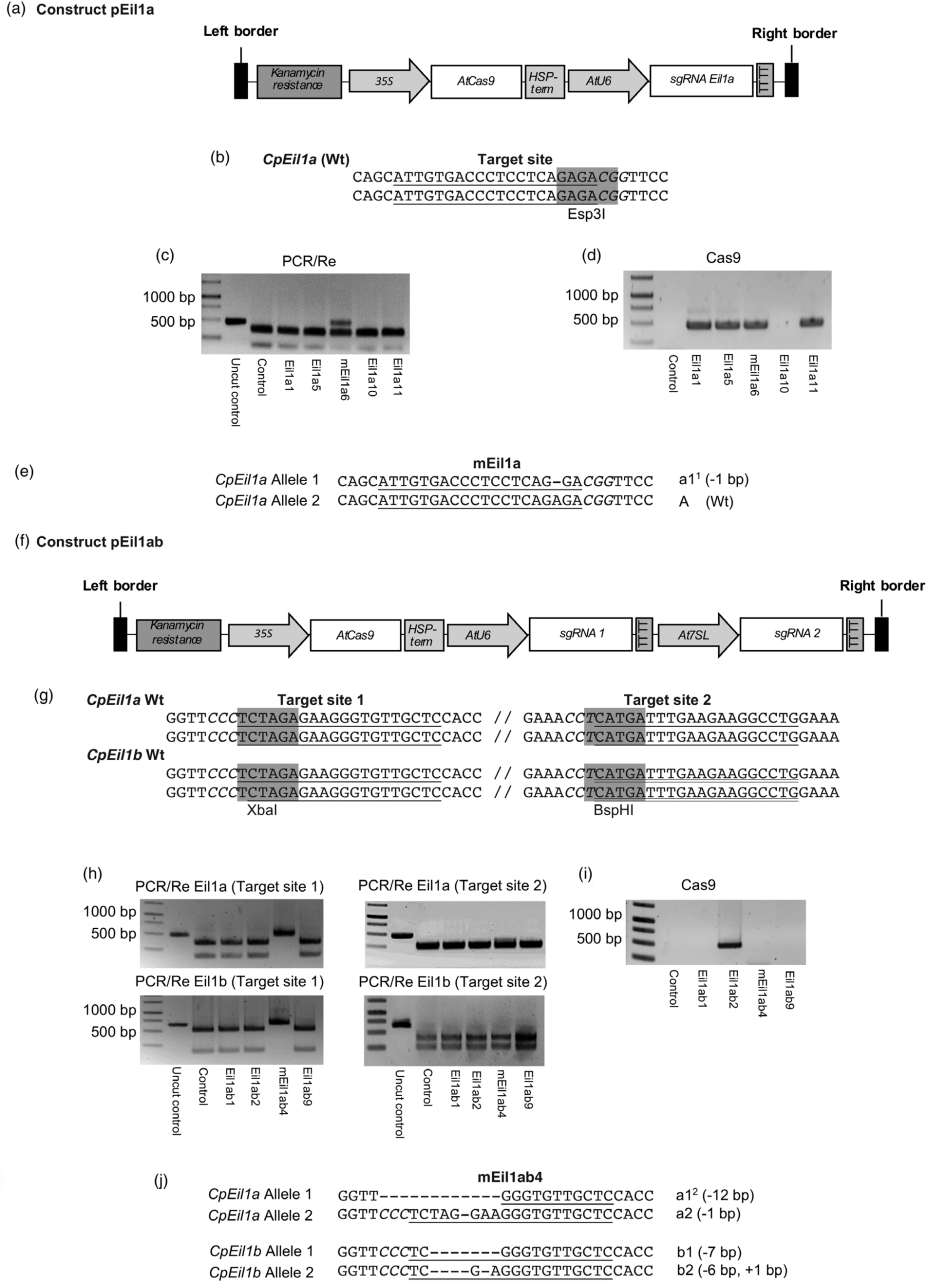
Mutation detection in the rooted *C. portenschlagiana* ‘PKMp11’ regenerants. (a) Schematic presentation of the T‐DNA in the pEil1a CRISPR/Cas9 vector. (b) Target and restriction site used to detect mutations in pEil1a‐rooted regenerants. (c) PCR/RE of the five rooted plants obtained after transformation with the pEil1a vector. (d) PCR detection of the presence of T‐DNA in these rooted plants, using primers for the Cas9 gene. (e) Sequence of the target site in the primary mutant mEil1a6. The mutation in allele 1 is named a1^1^ (1‐bp deletion). (f) Schematic presentation of the T‐DNA in the pEil1ab CRISPR/Cas9 vector. (g) Targets and restriction sites used to detect mutations in the pEilab‐rooted regenerants. (h) PCR/RE of the four rooted plants obtained after transformation with the pEil1ab vector. (i) PCR detection of the presence of T‐DNA in the rooted plants, using primers for the Cas9 gene. (j) Sequences of target sites 1 in the primary mutant mEil1ab4. The mutations in allele 1 and allele 2 of *CpEil1a* are named a1^2^ (12‐bp deletion) and a2 (1‐bp deletion) respectively. The mutations in allele 1 and allele 2 of *CpEil1b* are named b1 (7‐bp deletion) and b2 (6‐bp deletion and 1‐bp insertion) respectively. Target sites are underlined, and the PAM sequences are in italics. Restriction sites are highlighted in grey.

One construct (pEila) targeting *CpEil1a* and another construct (pEilab) targeting *CpEil1a* and *CpEil1b* were used for transformation of petioles from *in vitro*‐grown *C. portenschlagiana* ‘PKMp11’ plants. The pEil1a construct targeted a 20‐bp sequence in the core DNA‐binding domain of *CpEil1a* (Figure 2a, b; Figure S1), and the pEilab construct simultaneously targeted two 20‐bp sequences 86 bp apart in the core DNA‐binding domains of both *CpEil1a* and *CpEil1b* (Figure 2f, g). As expected from a previous *Agrobacterium* transformation study of *C. portenschlagiana* (Sriskandarajah *et al*., 2008), only few shoots were regenerated from callus formed on the infected explants. For the pEil1a construct, five rooted plants were generated from 1500 infected explants and these plants were tested for mutations. The PCR/RE test indicated a heterozygous mutation in one of the five rooted plants called mEil1a6 as half of the PCR product was digested by the restriction enzyme Esp3I (Figure 2c). Sequencing of the undigested band confirmed a mutation at the target site of 1‐bp deletion (Figure 2e). This mutated allele could be assigned to allele 1 and was named a1^1^. The mutation introduced a frame shift resulting in a premature stop codon in the mRNA, preventing the full translation of the proline‐rich region of the DNA‐binding domain and thus a non‐functional Eil1a TF (Figure S2). Subsequently, the presence of the CRISPR/Cas9 construct was investigated in the rooted plants, using PCR with primers for Cas9. Four of the five rooted plants, including the mutated plant mEil1a6, showed a Cas9 PCR fragment (Figure 2d).

For the pEil1ab construct, four rooted plants were generated from 1200 infected explants. The PCR/RE test indicated mutations in both alleles at target site 1 of *Eil1a* as well as *Eil1b* in plant mEil1ab4 as the PCR products remained undigested by the restriction enzyme XbaI (Figure 2h). In contrast, the PCR/RE test indicated no mutations at target site 2 for both *Eil1a* and *Eil1b* in any of the rooted plants as the PCR products were completely digested by BspHI (Figure 2h). Sequencing of the undigested PCR products at target site 1 showed biallelic mutations for both *Eil1a* and *Eil1b* (Figure 2j). For *Eil1a*, deletions of 12 bp and 1 bp were identified. These mutations could be assigned to allele 1 and allele 2 and were named a1^2^ and a2 respectively (Figure 2j). For *Eil1b*, mutations of 7 bp deletions and 6 bp deletions plus 1‐bp insert were identified. These mutations could be assigned to allele 1 and allele 2 of *Eil1b* and were named b1 and b2 respectively (Figure 2j). The frameshift mutations of a2, b1 and b2 introduced premature stop codons in mRNAs, preventing full translation of the proline‐rich region of the DNA‐binding domain, and the a1^2^ in frame deletion introduced amino acid changes and deletion of a short helical turn within the proline‐rich region of the DNA‐binding domain (Figure S2). This strongly indicates that all four mutated alleles of plant mEil1ab4 produce non‐functional Eil proteins.

The four rooted plants were subsequently investigated for the presence of the CRISPR/Cas9 construct. Cas9 was only detected in plant Eil1ab2 (Figure 2i). Due to lack of the Cas9 PCR product in the mutated plant mEil1ab4, this plant was further investigated using kanamycin resistance primers, but still no PCR product was obtained (data not shown). This clearly indicates that the CRISPR/Cas9 construct was only transiently expressed, inducing the mutations in *CpEil1a* and *CpEil1b* at target site 1.

### Ethylene tolerance of the primary mutants mEil1a6 and mEil1ab4

**Figure 3:**
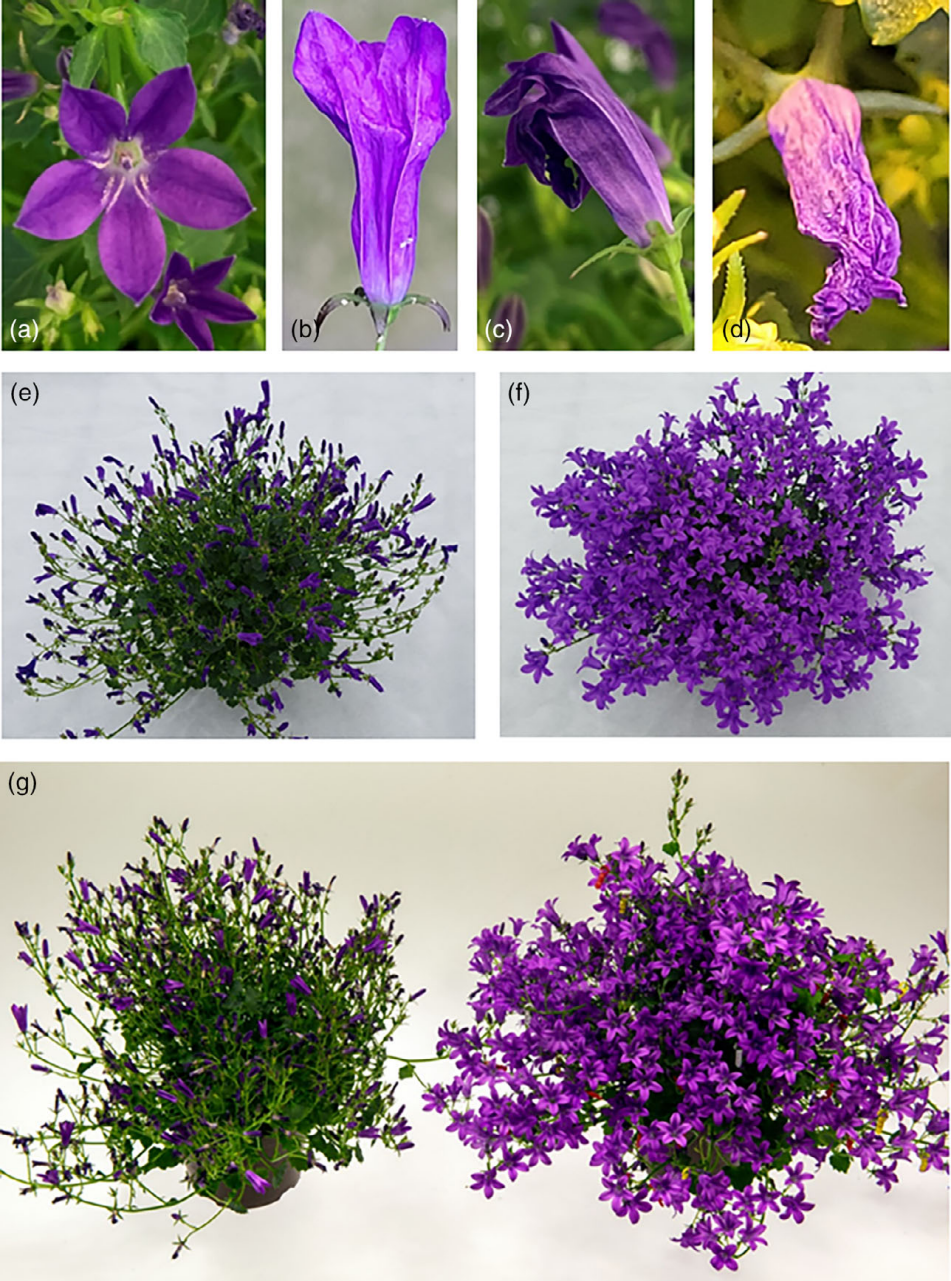
*C. portenschlagiana* flower senescence in response to exogenous ethylene treatments. (a‐d) Close‐up of flowers from non‐mutated *C. portenschlagiana* ‘PKMp11’ plants after 18 h of ethylene treatment at 0.25 ppm. (a) Non‐damaged flower. (b‐d) Ethylene damaged flowers. (e) Non‐mutated ‘PKMp11’ plant exposed to 0.25 ppm ethylene for 24 h. (f) Mutated mEil1ab4 plant exposed to 0.25 ppm ethylene for 24 h. (g) A non‐mutated ‘PKMp11’ (left) and a mutated mEil1ab4 plant (right) exposed to 5 ppm ethylene for 18 h. All ethylene exposures were performed at 20 °C.

Vegetatively propagated plants of the primary mutants were tested for flower ethylene tolerance at different ethylene concentrations. Flowers were treated for 18 h (h) at 20 °C and evaluated for ethylene tolerance 6–8 h after the treatments (Figure 3a–d). The number of ethylene‐senesced flowers out of the total number of flowers evaluated on each plant was counted and the percentage calculated. This percentage was used as an inverse measurement of ethylene tolerance, that is, the lower the percentage of senesced flowers, the higher the tolerance.

**Figure 4:**
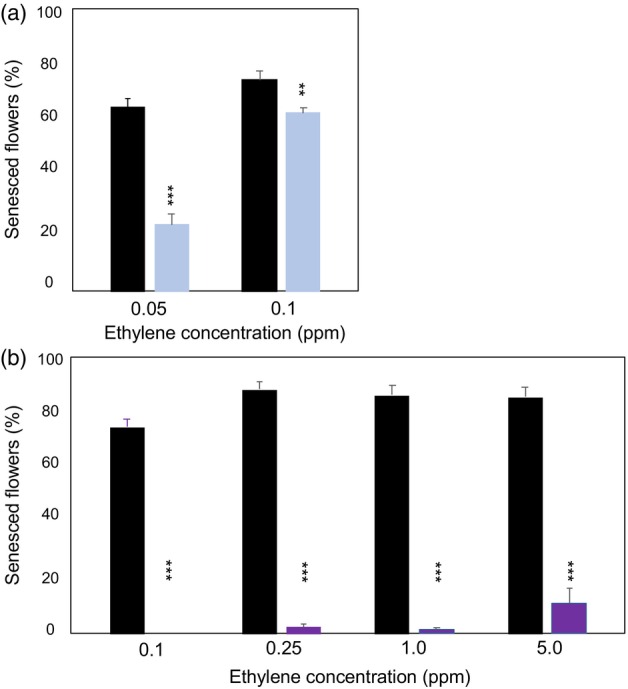
Ethylene sensitivity of primary mutants exposed to different ethylene concentrations. (a) Ethylene sensitivity of non‐mutated ‘PKMp11’ plants (black bars) and the primary mutant mEil1a6 plants (blue bars) treated with 0.05 and 0.1 ppm ethylene for 18 h at 20 °C. (b) Ethylene sensitivity of the non‐mutated ‘PKMp11’ plants (black bars) and the primary mutant mEil1ab4 plants (purple bars) treated with 0.1, 0.25, 1.0 and 5.0 ppm ethylene for 18 h at 20 °C. The percentage of senesced flowers was estimated 6–8 h after termination of the ethylene treatment. Each bar represents means of 3–21 vegetatively propagated plants, evaluating an average of 43 flowers per plant. The significance levels between the non‐mutated and the mutated plants were calculated using two‐tailed *t*‐tests. Error bars represent standard error. *Asterisks* indicate the significance level at **P < 0.01 and ***P < 0.001.

The mEil1a6 plants, mutated in only one *CpEil1a* allele, showed a significantly increased ethylene tolerance when treated with 0.05 and 0.1 ppm ethylene as compared to the non‐mutated ‘PKMp11’ plants (Figure 4a). When the flowers were evaluated 6–8 h after the 0.05 ppm ethylene treatment, an average of 23.7% senesced flowers was observed on mEil1a6 plants, whereas 65.1% senesced flowers were observed on the non‐mutated ‘PKMp11’ plants. At the 0.1 ppm ethylene treatment, the difference was much smaller but still significant with an average of 62.9% senesced flowers on mEil1a6 plants as compared to 74.8% senesced flowers on the non‐mutated ‘PKMp11’ plants (Figure 4a).

In contrast, no flowers on mEil1ab4 plants, mutated in all four *CpEil1* alleles, showed signs of senescence when treated with 0.1 ppm ethylene (Figure 4b). This strongly indicated that mutations in all four *CpEil1a* and *CpEil1b* alleles increased the ethylene tolerance more than the heterozygous mutation in only *CpEil1a*. The mEil1ab4 plants were further tested at higher ethylene concentrations of 0.25, 1.0 and 5.0 ppm (Figure 4b). Even at these ethylene concentrations, low mean senescence percentages of 2.3, 1.9 and 11.3%, respectively, were observed (Figure 3g; Figure 4b).

**Figure 5:**
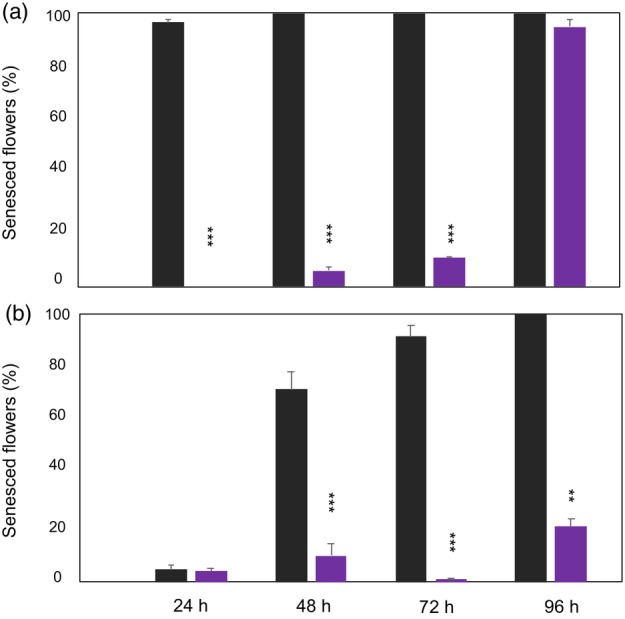
Ethylene sensitivity of the primary mutant mEil1ab4 exposed to 0.25 ppm ethylene for 24, 48, 72 and 96 h. (a) Results of treatments performed at 20 °C. (b) at 10 °C. Black and purple bars represent the non‐mutated ‘PKMp11’ plants and the mutated mEil1ab4 plants respectively. The percentage of senesced flowers was estimated 6 to 8 h after termination of the ethylene treatment. Each bar represents means of three to five vegetatively propagated plants where an average of 115 individual flowers was evaluated per plant. The significance levels between the non‐mutated and the mutated plants were calculated using two‐tailed *t*‐tests. Error bars represent standard errors. *Asterisks* indicate the significance level at **P < 0.01 and ***P < 0.001.

Ethylene tolerance is dependent on the temperature during ethylene exposure and the duration of the exposure. Thus, ethylene tolerance of non‐mutated and mutated mEil1ab4 plants was tested at 0.25 ppm ethylene at 20 °C and 10 °C for different exposure times (24, 48, 72 and 96 h). After treatment with 0.25 ppm ethylene at 20 °C for 24 h, none of the flowers on the mutated mEil1ab4 plants senesced, while an average of 96.5% of the flowers on non‐mutated plants senesced (Figure 3e,f; Figure 5a). When the ethylene exposure to 0.25 ppm was increased to 48, 72 and 96 h, the mean percentage of senesced flowers on mEil1ab4 plants increased to 5.9%, 10.4% and 95.1% respectively. In contrast, 100% of the flowers on the non‐mutated plants senesced at all these treatments. At 10 °C and 24 h exposure, the mutated plants showed almost the same mean percentage of senesced flowers (4.0%) as the non‐mutated plants (4.9%). At longer exposure times, the non‐mutated plants showed highly increased mean percentages of senesced flowers, that is, 71.2, 91.7 and 100% at 48 h, 72 h and 96 h respectively (Figure 5b). However, the mutated plants showed almost the same tolerance to the ethylene treatments at 10 °C and 20 °C when exposed for 48 and 72 h while the tolerance was greatly improved at 10 °C as compared to the treatment at 20 °C when exposed for 96 h. At this treatment the mean percentage of senesced flowers decreased from 95.1% at 20 °C to 20.7% at 10 °C (Figure 5b).

### Flower longevity and endogenous ethylene production during natural senescence

No significant difference was found between the longevity of ‘PKMp11’ and mEil1ab4 flowers (Figure S3a). Similarly, no significant differences in ethylene production were found within or between 8‐ and 11‐day‐old flowers from mEil1ab4 and ‘PKMp11’ plants (Figure S3b).

### Inheritance of the mEil1a6 and mEil1ab4 mutations to the next generations

The mEil1a6 plants were hand‐pollinated with the non‐mutated *C. portenschlagiana* blue clone ‘5628–21’. PCR/RE showed heterozygosity in F_1_ for a mutation which by sequencing was confirmed to be the same mutation (allele a^1^) as found in the parent plant (Figure S4), verifying that the mutation was inheritable. The mEil1a6 plant and its progeny showed a divergent phenotype. Compared to ‘PKMp11’, leaves were more wrinkled and yellowish. Flowering was delayed for 2 weeks and flowers were darker and larger than usual. They were almost male and female sterile, even though both pollen and ovules looked normal. Due to this, these mutated plants were not further investigated.

The mEil1ab4 plants on the other hand showed a phenotype very similar to ‘PKMp11’ but with flowering a few days earlier. Self‐pollination of the mEil1ab4 plants resulted in seven S_1_ seedlings. Moreover, mEil1ab4 plants were hand‐pollinated using the non‐mutated *C. portenschlagiana* blue clone ‘5628‐21’ as pollen donor. Here, we obtained 28 seedlings. The S_1_ and F_1_ plants were investigated for the presence of the mutations. New restriction sites caused by the mutations in mEil1ab4 allowed a clear PCR/RE distinction of the different mutated alleles in the progenies. To distinguish the mutation of allele a1^2^ (−12 bp) from allele a2 (−1 bp), a restriction with NlaIV of the PCR fragments was used. This restriction created two fragments (267 and 184 bp) and three fragments (267, 136 and 59 bp) in a1^2^ and a2 respectively (Table S1). To distinguish the mutation creating allele b1 (−7 bp) from allele b2 (−6, +1 bp), restriction digestion with XhoI was used. XhoI did not cut in b1 and created two fragments (486 and 144 bp) in b2 when the PCR fragment was digested with XhoI (Table S1).

As expected, S_1_ plants were either biallelic or homozygous for the mutated alleles a1^2^, a2, b1, b2 (Figure S5a), while all F_1_ plants were heterozygous for the mutations (Figure S5b). Each of the 28 F_1_ plants showed one of the four expected genotypes, and the number of plants within each genotype group was nine (Aa1^2^Bb1), six (Aa1^2^Bb2), four (Aa2Bb1) and nine (Aa2Bb2) (Figure S5b). The almost equal number of plants assigned to each genotype indicates that there is no linkage between *CpEil1a* and *CpEil1b* and that the two genes are located on different chromosomes or far apart on the same chromosome. Furthermore, these numbers also indicate that neither of the mutations were lethal during seed development and germination.

Next, F_1_ plants were self‐pollinated by hand to get F_1_ progenies with more combinations of mutated and non‐mutated *CpEil1* alleles. The 45 F_2_ plants obtained were analysed by PCR/RE as described above (Figure S6). Six of the 45 F_2_ plants showed cross‐pollination between the F_1_ groups (Table S2). The reason for this is unknown but these six plants were also included in the further studies.

### Ethylene tolerance of the progenies from the primary mutant mEil1ab4

**Figure 6:**
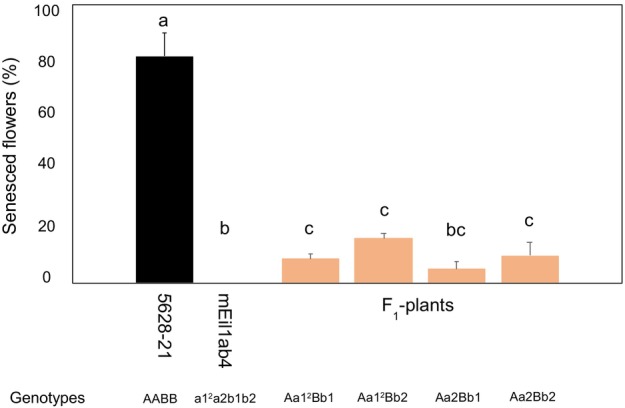
Ethylene sensitivity of the parents and F_1_ plants exposed to 0.1 ppm ethylene. The percentage of senesced flowers of the parents (blue clone ‘5628‐21’ and the mEil1ab4 primary mutant) and F_1_ plants (orange bars) was estimated 6–8 h after termination of the ethylene treatment for 18 h at 20 °C. Each bar represents means of 3–13 individual plants, evaluating an average of 31 flowers per plant. Error bars represent standard errors. The significance differences between the genotypes are indicated with letters above each column and values with the same letter(s) are not significantly different (*P* < 0.05).

F_1_ flowering plants within each genotype group were evaluated for ethylene tolerance at 0.1 ppm ethylene together with their parents (‘5628–21’ and the primary mutant mEil1ab4). The mean senescence percentages of F_1_ plants of the four genotypes Aa1^2^Bb1, Aa1^2^Bb2, Aa2Bb1 and Aa2Bb2 were 8.8, 16.6, 5.8 and 9.9%, respectively, and no significant difference was found between the four F_1_ genotypes (Figure 6), indicating that the different mutations contribute equally to the increased ethylene tolerance. The mean senescence percentages of F_1_ plants within the four genotype groups were all significantly lower than the parent ‘5628–21’ with a mean senescence percentage of 81.5% and—except for the genotype group Aa2Bb1—significantly higher than the parent mEil1ab4 with a senescence percentage of 0%.

**Figure 7:**
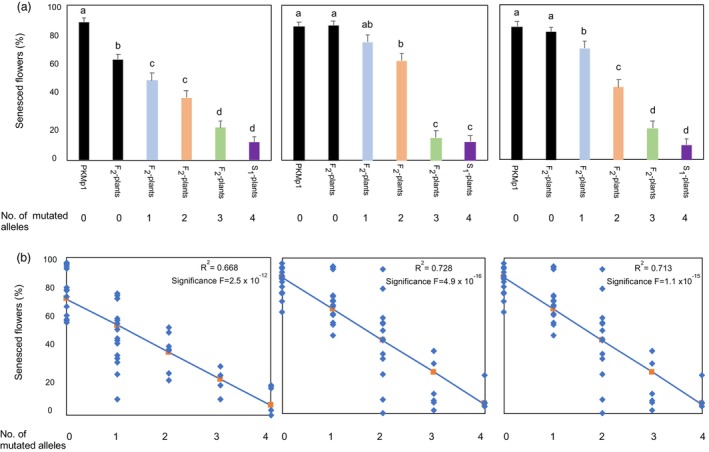
Ethylene sensitivity of the F_2_ plants divided into groups with zero, one, two or three mutated alleles and the S_1_ plant group with four mutated alleles. (a) Plants within each group and non‐mutated ‘PKMp11’ plants treated with 0.25, 1.0 or 5.0 ppm ethylene for 18 h at 20 °C. The percentage of senesced flowers was estimated 6–8 h after termination of the ethylene treatment. Each bar represents means of 4–17 individual plants with an average of 37 flowers evaluated per plant. The significance differences between the groups are indicated with letters above each bar, and values with the same letter(s) are not significantly different (*P* < 0.05). Error bars represent standard errors. (b) Linear regression analysis between the number of mutated alleles and the percentage of senesced flowers for each ethylene treatment. Each point represents the results from individually evaluated plants. Plants with 0 mutated alleles represent results obtained with the non‐mutated ‘PKMp11’ plants as well as the F_2_ plants without mutations. *R*‐squared values and significant levels of the linear regression are indicated for each ethylene treatment.

Subsequently, flowering plants of the 45 F_2_ plants, the seven S_1_ plants and the non‐mutated ‘PKMp11’ were investigated for ethylene tolerance at 0.25 ppm, 1.00 ppm and 5.00 ppm ethylene (Figure 7a). As none of the mutated alleles contributed more or less to the ethylene tolerance, F_2_ plants with different combinations of mutated alleles were divided into four groups each with zero, one, two or three mutated alleles. The S_1_ plants were grouped into one group with four mutated alleles (Figure 7a). The different combinations of alleles within each group and the number of plants tested at each ethylene concentration are listed in Table S2.

The 0.25 ppm ethylene treatment showed a significant difference between the non‐mutated ‘PKMp11’ (control of the S_1_ plants) and the non‐mutated plants obtained from the F_2_ crosses (control of the F_2_ plants). Still, plants with 1, 2, 3, 4 mutated alleles all showed significantly more ethylene tolerance than both controls (Figure 7a). At the 1.00 ppm and 5.00 ppm ethylene treatments there was no significant difference between the non‐mutated ‘PKMp11’ and the non‐mutated plants obtained from the F_2_ crosses. As observed for plant mEil1a6 at 0.1 ppm ethylene, these data show that even when only one *Eil1* allele was mutated, an increase in ethylene tolerance was achieved, although this increase was not significant at 1.00 ppm ethylene (Figures 4a and 7a). When two *Eil1* alleles were mutated, a significant increase in ethylene tolerance was observed in all three ethylene treatments, as was also observed at 0.1 ppm in the F_1_ plants (Figures 6 and 7a). Although the high increase in ethylene tolerance of plants with three mutated *Eil1* alleles was not significantly different from plants with four mutated alleles in any of the three ethylene treatments, the percentage of senesced flowers was consistently lower in plants with four mutated alleles at all three ethylene treatments. This indicates an additive effect of the mutated alleles, and that maximum ethylene tolerance is obtained when all four *Eil1* alleles are mutated. The additive effect of the number of mutated *Eil1* alleles on ethylene tolerance was confirmed by significant regression analysis between the number of mutated alleles and the mean percentage of senesced flowers which showed R^2^ values of 0.67, 0.73 and 0.71 for plants treated at 0.25, 1.00 and 5.00 ppm ethylene respectively (Figure 7b). This indicates that an average of 70% of the variation in senescence percentages at all three ethylene treatments can be explained by the number of mutated *Eil1* alleles present in the plants.

## Discussion

Early senescence of climacteric flowers and fruits induced by ethylene exposure during transportation and storage is a major problem (Ma *et al*., 2018; Paul *et al*., 2012; Van Doorn and Woltering, 2008; Woltering and Van Doorn, 1988). In order to identify handles for controlling tolerance against exogenous ethylene, the DNA sequences encoding the proline‐rich regions of the DNA‐binding domain of *C. portenschlagiana* CpEil1a and CpEil1b were mutated using CRISPR/Cas9. This generated two mutants, mEil1a6 and mEil1ab4, containing a mutated allele in the *Eil1a* (a1^1^) and two mutated alleles in both *Eil1a* (a1^2^a2) and *Eil1b* (b1b2) respectively. Four mutations resulted in amino acid frameshifts and stop codons in the mRNA. The fifth (−12 bp) deletion mutation in allele a1^2^ of the mEil1ab4 plant resulted in a four amino acid deletion and one amino acid change. This mutation disrupts a short helical turn within the proline‐rich region, important for protein function (Song *et al*., 2015). Also, the first residue in this short helical turn (leucine 206 in *AtEIN3*) is important for hydrophobic interactions stabilizing the protein conformation (Yokotani *et al*., 2003). This strongly indicates that all mutated Cpeil1 proteins are no longer able to bind to their recognition motifs in the promoters of the ethylene‐regulated genes.

Vegetatively propagated plants of both mEil1a6 and mEil1ab4 showed an increased ethylene tolerance when treated with 0.1 ppm ethylene for 18 h at 20 °C. The increase was much higher for mEil1ab4 plants, which developed no senesced flowers at this treatment. Moreover, mEil1ab4 plants showed very high ethylene tolerance to treatments with ethylene concentrations of 0.25, 1.0 and 5.0 ppm for 18 h at 20 °C. This clearly shows that the *CpEil1* genes are deeply involved in the response to exogenous ethylene and that mutations in all four alleles provide a much higher tolerance to exogenous ethylene than a mutation in only one allele. In contrast, flowers of mutated mEil1ab4 and non‐mutated ‘PKMp11’ showed no significant differences in longevity or endogenous ethylene production during senescence, indicating that CpEil1 is not involved in age‐dependent senescence of flowers. This is supported by studies in carnation where lines bred for increased flower longevity showed lower levels of endogenous ethylene but not lower sensitivity towards exogenous ethylene (Onozaki *et al*., 2006). Flower longevity and sensitivity towards exogenous ethylene appear to be controlled by different mechanisms.

Besides the concentration of ethylene, tolerance to exogenous ethylene is also dependent on exposure time and temperature (Macnish *et al*., 2004; Serek *et al*., 2006). Thus, vegetatively propagated mEil1ab4 plants were further investigated for ethylene tolerance under longer exposure periods. When plants were treated with 0.25 ppm ethylene for 24, 48 and 72 h at 20 °C, a significant and much higher tolerance to ethylene was found in mutated mEil1ab4 plants as compared to non‐mutated plants. However, 96 h of continuous ethylene treatment caused a sudden decrease in ethylene tolerance, resulting in 95% senesced flowers. The flower senescence was definitely induced by the continuous exogenous ethylene treatment as non‐mutated plants placed in the ethylene cabinet for 96 h without ethylene treatment did not show any senesced flowers. Long‐time exposure to exogenous ethylene potentially simulates abiotic stress that induces expression of other *CpEIN3/Eils*. Investigations of *cis*‐acting elements in the promoters of *EIN3/Eils* in different species indicate that some *EIN3/Eils* are co‐regulated by signalling molecules involved in abiotic stress (He *et al*., 2020; Jyoti *et al*., 2021). Further studies are needed to uncover the effects of very long exposure times and temperature on overall *CpEIN3/Eils* expression. Currently, low temperatures are known to slow down all biochemical processes including ethylene responses (Macnish *et al*., 2004; Serek *et al*., 2006; Woltering and Harkema, 1987). This was also observed in the current experiments as mEil1ab4 plants exposed to 96 h ethylene at 10 °C showed a significant and much lower ethylene sensitivity of 21% as compared to 95% at 20 °C.

As opposed to plant mEil1ab4, which showed a phenotype very similar to the non‐mutated plants, plant mEil1a6 showed a divergent phenotype indicating off‐target mutations or somaclonal variation induced during tissue culture. For this reason, further analysis with mEil1a6 was not performed. Still, variation in the number of functional and non‐functional *CpEil1* alleles could be generated through crossing of the mEil1ab4 plants, mutated in all four *Eil1* alleles, with plants of a non‐mutated clone. The segregation pattern of *CpEil1a* and *CpEil1b* among these progenies showed that the two *CpEil1* genes were unlinked, allowing all possible allele combinations between the functional and the non‐functional *CpEil1* alleles. Sixteen different combinations of functional and non‐functional *Eil* alleles were present among the 45 progenies obtained from the F_1_ plants. Additionally, seven plants with six different combinations of the mutated *Cpeil1* alleles were obtained from self‐pollination of the mEil1ab4 plant. As all mutated alleles seem to contribute equally to the ethylene tolerance, a panel of lines with zero, one, two, three or four mutated eil alleles was generated. The additive effect of the *CpEil1a* and *CpEil1b* alleles became very clear when these plants were treated with 0.25, 1.0 or 5.0 ppm ethylene. Even flowers on plants with only one mutated *Cpeil1* allele showed increased tolerance to ethylene treatments of 0.25, 1.0 and 5.0 ppm, and there was a significant correlation between the number of mutated alleles and the percentage of senesced flowers in all three ethylene treatments. Thus, our studies uncovered a significant allele dosage effect of the number of functional *Eil1* alleles on the flower ethylene tolerance.

*CpEil1a* and *‐1b* are very similar in sequence and therefore likely arose by duplication. Duplicated, unlinked *Eils* were also found in several other species, including pear and banana (Cao *et al*., 2017; Jourda *et al*., 2014). Expression profiles of duplicated pear *PbEIL* and banana *MaEil* genes showed different expression patterns in different tissues, indicating sub‐functionalization after duplication (Cao *et al*., 2017; Jourda *et al*., 2014). In the current study, the function of CpEil1a and CpEil1b seems to be similar in ethylene‐induced flower senescence. However, they might have other functions not observed under the conditions used in the present study. The total knockout of all *CpEil1a* and *CpEil1b* alleles might therefore influence other important functions of these TFs such as responses to biotic and abiotic stress. Knockout of only one *CpEil1a* and one *CpEil1b* allele might be more beneficial as it reduces the ethylene‐induced flower senescence considerably, while other important functions of CpEil1 are still retained.

The allelic dosage effect observed in the present study constitutes a handle for regulating ethylene tolerance in plants showing early senescence induced by exogenous ethylene. The allelic dosage effect handle can be especially advantageous in species with more than one *Eil* gene having the same function during early senescence. Here, a gradual regulation of ethylene sensitivity is likely to be feasible. Such species include petunia where two *PhEil* genes are involved in flower senescence (Liu *et al*., 2016), carnation with three *DcEil* genes, all highly expressed in petals after exogenous ethylene exposure (Iordachescu and Verlinden, 2005), tomato where three *LeEil* genes are involved in fruit ripening (Fu *et al*., 2005; Tieman *et al*., 2001) and pear with three *PbEIL* genes highly expressed during late fruit ripening (Cao *et al*., 2017).

In summary, the results of this study provide insights into the roles of the *CpEil1a* and *CpEil1b* genes in regulating the response towards exogenous ethylene in *C. portenschlagiana* and suggest focus on them as the key handles for a gradual modulation of the sensitivity towards exogenous ethylene in plants.

## Experimental procedures

### CRISPR/Cas9 constructs for plant transformation

We used *C. portenschlagiana* ‘PKMp11’ (sold as ‘Blue GET MEE®’) for cloning of gene fragments. Total RNA was purified from flower buds using the Direct‐zol RNA Purification Kit (Zymo‐Research, Denmark). Superscript IV (Thermo Fisher Scientific, Denmark) was used to obtain 3´RACE cDNAs with the anchored 3RACE_rev primer (Table S3). Two *CpEil1a* alleles of 2262 bp were cloned from the 3′RACE cDNAs using the Cp_EIL1b_fw and the Tracer_3RACE_rev primers (Figure S1, Table S3). Both alleles were cloned from flower buds. For *CpEil1b*, primers Cp_EIL1b_fw and Cp_EILb_rev (Figure S1, Table S3) were used to clone two alleles of 924 bp from cDNA obtained from flower buds. Two CRISPR/Cas9 constructs named pEil1a and pEil1ab were designed. pEil1a targeted a 20‐bp sequence specific for only the *CpEil1a* genomic sequence (Figure S1). pEil1ab targeted two 20‐bp sequences 86 bp apart in both *CpEil1a* and *CpEil1b* as the two 20‐bp spacer sequences were identical in the *CpEil1a* and *CpEil1b* genomic sequences (Figure S1).

Vector constructs were assembled using the modular vector system designed for Golden Gate cloning (Čermák *et al*., 2017). All plasmids used were kindly provided by the Dan Voytas Lab. For the pEil1a vector, the following A, B, C and transformation backbone plasmids were used for Golden Gate cloning: pMOD_A0101 (Addgene #90998, containing *35S*:*AtCas9*), pMOD_B2515 (Addgene #91072, containing *AtU6*:gRNA), pMOD_C0000 (Addgene #91081, containing an empty plasmid used as placeholder) and pTRANS_220 (Addgene #91113, containing *35S:KanR* in the T‐DNA as plant selection marker). For the construction of the pEil1ab vector, the protocols and plasmids used were the same except that the plasmid pMOD_C2516 (Addgene #91084, containing *At7SL*:gRNA) was used instead of pMOD_C0000. The final vector constructs of pEil1a and pEil1ab are shown in Figure 2a and 2f respectively.

The *Agrobacterium* transformation vectors were subsequently transformed into the *Agrobacterium tumefaciens* strain AGL1, using the freeze/thaw method. Positive clones were selected on medium with 25 μg/mL rifampicin and 50 μg/mL kanamycin.

### Plant transformation

Two‐ to three‐month‐old (2–3 cm high) *in vitro‐*grown *C. portenschlagiana* ‘PKMp11’ plants were provided by Vitroform, Denmark and used as donor material for *Agrobacterium* transformation. The plants were grown at 23 °C under short‐day light conditions (9 h/15 h light/dark) to avoid flower induction. Petioles were dissected into 0.7 cm long sections and infected with *Agrobacterium* (harbouring pEil1a or pEil1ab) by submerging them for 30 min under constant shaking at 100 rpm in an overnight *Agrobacterium* culture diluted to OD_600_ = 0.5. The infected explants were incubated on co‐culture medium in the dark at 23 °C for 48 h (Table S4). Then the explants were transferred to selection medium containing 300 mg/L timentin (ticarcillin disodium/clavulanate potassium (Duchefa Biochemie, Haarlem, NL)) and 100 mg/L kanamycin (Duchefa Biochemie, Haarlem, NL), incubated at 23 °C under dim light (9 h/15 h light/dark) and sub‐cultured every 4 weeks. Regenerated shoots started to appear after 3–5 months on the selection medium. When shoots were ~ 1 cm long, they were moved to rooting medium (Table S4) and incubated at 23 °C under light (9 h/15 h light/dark) until root formation. In order not to kill all shoots, the kanamycin concentration in the rooting medium was reduced to 50 mg/L. Rooted shoots were potted in soil and grown at 9 h/15 h light/dark at 18 °C in the greenhouse. When the plants had a rosette diameter of at least 5 cm they were vegetatively propagated by cuttings. Plants were kept in short‐day conditions and propagated regularly for production of new stock/mother plants and flowering plants for ethylene testing.

### Molecular analysis of rooted regenerants

Leaves were collected from the rooted plants after transfer to soil, and genomic DNA was isolated using the FastDNA™ kit (MP Biomedicals) or by the phenol/chloroform method. PCR/restriction (PCR/RE) was used for initial detection of mutations. For both the pEil1a‐ and pEil1ab‐infected plants, a PCR product of 463 bp containing all three target sites in *Eil1a* was amplified using the primers tgEil1aF and tgEil1aR (Table S3). For pEil1ab‐infected plants, a PCR product of 625 bp containing the two target sites in *CpEil1b* was amplified using the tgEil1bF and tgEil1bR primers (Table S3).

For pEil1a‐infected plants, the PCR products were digested o/n with the restriction enzyme Esp3I having a recognition site predicted to be disrupted by Cas9 (Figure 2b). This restriction enzyme cuts the wild‐type PCR product into two fragments of 125 and 338 bp respectively (Figure 2c). Here we identified the heterozygous primary *CpEil1a* mutant plant mEil1a6 (Figure 2e).

For pEil1ab‐infected plants, the PCR products were digested with the restriction enzymes XbaI and BspHI having recognition sites predicted to be disrupted by Cas9 at target site 1 and 2 respectively (Figure 2g; Table S1). Without mutations in *Eil1a* at the XbaI and BspHI sites, the enzymes cut the 463‐bp *Eil1a* PCR product into 143 and 320 bp fragments and 215 and 248 bp fragments respectively (Figure 2h). Without *Eil1b* mutations at the XbaI and BspHI recognition sites, the enzymes cut the *Eil1b* PCR product into 143 and 492 bp fragments and 222 and 413 bp fragments respectively (Figure 2h). Here we identified the primary mutant mEil1ab4 with biallelic mutations at target site 1 in both the *CpEil1a* gene and the *CpEil1b* gene (Figure 2h).

Undigested bands were gel purified and cloned using the Zero Blunt TOPO PCR cloning kit (Invitrogen). For each PCR product, 4–20 clones were sequenced. The first part of the core DNA‐binding domain of the protein sequence, where the mutations were induced, was aligned using Clustal Omega (Madeira *et al*., 2019) to identify where a stop codon in mRNA or a helical obstruction was introduced by the mutations (Figure S2).

The presence of the CRISPR/Cas9 insert was analysed using primers for Cas9 (Table S3) amplifying a 467‐bp long fragment.

### Molecular analysis of progenies obtained from the primary mutants

Self‐pollination by hand of the primary vegetatively propagated mutants was attempted but without success in mEil1a6 plants and with moderate success in mEil1ab4 plants, resulting in only seven mEil1ab4 S_1_ seedlings. The mEil1a6 and mEil1ab4 plants were also crossed by hand‐pollination to a non‐mutated blue *C. portenschlagiana* clone called ‘5628‐21’. Progenies obtained from self‐pollinations and out‐crossings (S_1_ and F_1_ plants) were analysed for mutations by PCR/RE as described above. Moreover, new restriction enzyme sites created by the mutations were used for PCR/RE to distinguish the different allele combinations in the seven S_1_ and 28 F_1_ plants obtained (Table S1). The PCR/RE results in eight of the F_1_ plants were confirmed by sequencing.

F_1_ plants obtained from mEil1ab4 were further self‐pollinated by hand to obtain F_1_ progenies with different combinations of non‐mutated and mutated alleles. The 45 F_2_ plants obtained were analysed for mutations and allele combinations by PCR/RE, using the new restriction enzyme sites created by the mutations (Table S1).

### Testing ethylene tolerance of the primary mutants mEil1a6 and mEil1ab4

For flower induction, plants were transferred to long‐day conditions (16 h/8 h light/dark) 7 weeks after propagation. Six to seven weeks later, the flowering plants were ready for ethylene tolerance screening. For mEil1a6, nine plants were evaluated for ethylene sensitivity while for mEil1ab4, 44 plants were evaluated for ethylene sensitivity. The ethylene treatments took place in an airtight, temperature controlled, dark chamber (2.4 × 2.7 × 3 m, equivalent to 19 440 litres). Specific concentrations of ethylene within the chamber were obtained by injecting gaseous ethylene at the start of the exposure period. Concentrations of 0.05, 0.1, 0.25, 1.0 or 5.0 ppm, a temperature of 20 °C and an exposure time of 18 h were used. In one experiment, mutated mEil1ab4 plants were exposed to 0.25 ppm ethylene for 24, 48, 72 or 96 h at two different temperatures (10 °C or 20 °C). After the ethylene treatments, plants were transferred to the greenhouse. To get a direct measure of the effect of the different ethylene treatments, the flowers were evaluated 6–8 h later. The number of ethylene‐senesced flowers out of the total number of flowers evaluated on each plant was counted and the percentage of senesced flowers was calculated and used as an inverse measurement of ethylene tolerance, that is, the lower the percentage of senesced flowers, the higher the tolerance.

### Testing ethylene tolerance of F_1_ plants obtained from the primary mutant mEil1ab4

The 28 F_1_ plants obtained from the crosses between the primary mutant mEil1ab4 plants and the clone ‘5628‐21’ were tested at 0.1 ppm ethylene together with their parents.

### Testing ethylene tolerance of S_1_ and F_2_ plants obtained from the primary mutant mEil1ab4

The seven S_1_ plants and the 45 F_2_ plants together with ‘PKMp11’ were tested at 0.25 ppm, 1 ppm and 5 ppm ethylene. Each plant was tested for ethylene tolerance one to five times with 5–15 days in between so that new flowers were developed between each test and only new flowers were included in the estimation of ethylene tolerance.

### Flower longevity and endogenous ethylene level

The effect of the mutated Cp*eil1* alleles on flower longevity was analysed in flowers of ‘PKMp11’ and mEil1ab4 plants grown at 20 °C (16 h/8 h light/dark). Individual flowers were labelled at the bud stage and at flower opening. Time from flower opening to signs of senescence was recorded for 10 flowers on each plant. The mean number of days between flower opening and senescence was used as a measure of flower longevity. For measuring flower endogenous ethylene levels, individual flowers on three ‘PKMp11’ and three mEil1ab4, without major signs of senescence 8 and 11 days after opening were harvested. Two times four flowers from each plant were weighed and sealed in a serum bottle with a total volume of 58.5 mL. Ethylene released over 48 h was quantified by dynamic headspace analysis, using purified air (Supelpure, Supelco) of the serum bottles. The concentration of ethylene was measured by chemical ionization mass spectrometry (PTR‐MS) with O_2_^+^ as reagent ion. The method is based on Cappellin *et al*. (2014) with m/z 28 used to detect ethylene. A modified quadrupole PTR‐MS (Ionicon, Innsbruck, Austria) was used with a sampling frequency of 0.4 Hz and a fixed headspace flow of 85 mL/min. The total mass of ethylene was analysed by integrating the concentration‐time profile, using the trapezoid rule. Each sample was measured for 4.25 min, which covered >99% of the mass released. The detection limit of the method was determined to be 4 ppb based on repeated blank measurements. Calibration was based on dilutions of a certified ethylene standard of 10 ppm (Linde Gas) at concentrations from 10 ppb to 1.7 ppm (slope uncertainty: 2%, *R* ^2^ = 0.994).

### Statistics

The *t*‐test for unequal variance was employed to test significant differences in ethylene tolerance of all comparisons performed in this study. Relationships between the number of mutated alleles and the senescence percentages were analysed by regression analysis. All statistical tests were performed in Excel.

#### Accession numbers

The sequences of the *CpEIil1* alleles were submitted to the GenBank database under accession numbers *CpEil1a*_allele 1 (OM925995), *CpEil1a*_allele 2 (OM925996), *CpEil1b*_allele 1 (OM925997) and *CpEil1b*_allele 2 (OM925998). The address of GenBank is https://www.ncbi.nlm.nih.gov.

## Author contributions

HBP and KK designed the project. GD and DP‐S made the constructs. IBH and CRI conducted the transformations, the molecular analysis and analysed the data. KK tested the plants for ethylene tolerance. AF conducted the measurements of endogenous ethylene production in flowers. IBH, CRI and HBP wrote the initial manuscript, which was carefully revised by KK, GD, DP‐S and AF. All authors read and approved the final manuscript.

## Conflict of interest

The authors declare no conflict of interest.

## Supporting information


**Figure S1** Nucleotide alignment of the *CpEil1a* (allele 1 and allele 2) and *CpEil1b* (allele 1 and allele 2) isolated from *C. portenschlagiana* ‘PKMp11’

**Figure S2** Alignment of the first part of the core DNA‐binding domain of Ein3/Eil proteins

**Figure S3** Flower longevity and endogenous ethylene production of non‐mutated ‘PKMp11’ and mutated mEil1ab4 flowers

**Figure S4** Detection of mutations by PCR/RE in *CpEil1a* of the two F_1_ plants obtained after cross‐pollination of mEil1a6 with the blue clone ‘5628‐21’

**Figure S5** Detection of mutations by PCR/RE in *CpEil1a* and *CpEil1b* of the progenies from the primary mutant mEil1ab4 (a) of the seven S_1_ plants obtained after self‐pollination and (b) of the 28 F_1_ plants obtained after cross‐pollination with the blue clone ‘5628‐21’

**Figure S6** Detection of mutations by PCR/RE in *CpEil1a* and *CpEil1b* of the 45 F_2_ plants

**Table S1** Fragments created by PCR/RE in the primary mutant mEil1ab4 and progenies

**Table S2** The different combinations of mutated and non‐mutated alleles identified in F_2_ plants and S_1_ plants

**Table S3** PCR primers used in this study

**Table S4** Composition of media used for *Agrobacterium tumefaciens*‐mediated transformation of *C. portenschlagiana* ‘PKMp11’

## Data Availability

The data of this study are available from the corresponding author upon request.
