# Supplementary material for: CRISPR/Cas9‐mediated mutation of Eil1 transcription factor genes affects exogenous ethylene tolerance and early flower senescence in Campanula portenschlagiana
Source: Plant Biotechnol J. 2023 Oct 12;22(2):484–96. doi: 10.1111/pbi.14200 (PMC10826993; doi:10.1111/pbi.14200)
Supplement: Supplementary file 1 — Figure S1 Nucleotide alignment of the CpEil1a (allele 1 and allele 2) and CpEil1b (allele 1 and allele 2) isolated from C. portenschlagiana ‘PKMp11’ [file PBI-22-484-s007.docx]

CpEil1a_allele1 GAGCTAGAGAGGAGGATGTGGAGAGACAGAATGCTTCTGAGGCGGTTAAAAGAACAAAAT 60

CpEil1a_allele2 GAGCTAGAGAGGAGGATGTGGAGAGACAGAATGCTTCTGAGGCGGTTAAAAGAACAAAAT 60

CpEil1b_allele1 ------------------------------------CTGAGGCGGTTAAAAGAACAAAAC 24

CpEil1b_allele2 ------------------------------------CTGAGGCGGTTAAAAGAACAAAAC 24

***********************

CpEil1a_allele1 AAAGGCAAAGAAGGAGTTGTTGACAATGCCAAGCAACGTCAGTCACAAGAGCAAGCCCGT 120

CpEil1a_allele2 AAAGGCAAAGAAGGAGTTGTTGACAATGCCAAGCAACGTCAGTCACAAGAGCAAGCCCGT 120

CpEil1b_allele1 AAAGGCA---AAGAAGGAGTTGACAATGCCAAGCAACGTCAGTCTCAAGAACAAGCACGT 81

CpEil1b_allele2 AAAGGCA---AAGAAGGAGTTGACAATGCCAAGCAACGTCAGTCTCAAGAACAAGCACGT 81

******* *** ** ************************** ***** ***** ***

CpEil1a_allele1 AGAAAGAAGATGTCCCGAGCACAAGATGGTATACTCAAGTACATGCTAAAAATGATGGAA 180

CpEil1a_allele2 AGAAAGAAGATGTCCCGAGCACAAGATGGTATACTCAAGTACATGCTAAAAATGATGGAA 180

CpEil1b_allele1 AGAAAGAAGATGTCCCGGGCACAAGATGGTATTCTCAAGTACATGC**C**AAAAATGATGGAA 141

CpEil1b_allele2 AGAAAGAAGATGTCCCGGGCACAAGATGGTATTCTCAAGTACATGC**T**AAAAATGATGGAA 141

***************** ************** ************* *************

CpEil1a_allele1 GTTTGTAAAGCTCAGGGTTTTGTGTATGGGATCATCCCTGAGAACGGAAAGCCTGTCAGT 240

CpEil1a_allele2 GTTTGTAAAGCTCAGGGTTTTGTGTATGGGATCATCCCTGAGAACGGAAAGCCTGTCAGT 240

CpEil1b_allele1 GTTTGTAAAGCTCAGGGTTTTGTGTATGGGATCATCCCCGAGAAAGGAAAGCCTGTCAGT 201

CpEil1b_allele2 GTTTGTAAAGCTCAGGGTTTTGTGTATGGGATCATCCCCGAGAAAGGAAAGCCTGTCAGT 201

************************************** ***** ***************

CpEil1a_allele1 GGGGCCTCTGACAATCTTCGTGCGTGGTGGAAGGAGAAAGTACGGTTTGATCGCAATGGC 300

CpEil1a_allele2 GGGGCCTCTGACAATCTTCGTGCGTGGTGGAAGGAGAAAGTACGGTTTGATCGCAATGGC 300

CpEil1b_allele1 GGAGCCTCTGACAATCTTCGTGCTTGGTGGAAGGAGAGAGTACGGTTTGATCGAAACGGC 261

CpEil1b_allele2 GGAGCCTCTGACAATCTTCGTGCTTGGTGGAAGGAGAGAGTACGGTTTGATCGAAACGGC 261

** ******************** ************* *************** ** ***

CpEil1a_allele1 CCCGCTGCTATAGCCAAGTATCAGGCTGAC**CATTCAGCCCCTGGGAAGAG**CGAGGACTGC 360

CpEil1a_allele2 CCCGCTGCTATAGCCAAGTATCAGGCTGAC**CATTCAGCCCCTGGGAAGAG**CGAGGACTGC 360

CpEil1b_allele1 CCGGCTGCAATAACCAAGTACCAGGCAG**ACCATTCAGTCCCCGGTG**AGAGTGAGGACTGC 321

CpEil1b_allele2 CCGGCTGCAATAACCAAGTACCAGGCAG**ACCATTCAGTCCCCGGTG**AGAGTGAGGACTGC 321

** ***** *** ******* ***** ********** *** ** **** *********

CpEil1a_allele1 TGTACGATGGCCTCCACGCCCCACACATTGCAAGAGCTTCAGGACACCACTCTTGGCTCA 420

CpEil1a_allele2 TGTACGATGGCCTCCACGCCCCACACATTGCAAGAGCTTCAGGACACCACTCTTGGCTCA 420

CpEil1b_allele1 TGTATGGTTGCCTCCACGCCTCACACACTTCAAGAGCTTCAGGACACGACTCTTGGCTCA 381

CpEil1b_allele2 TGTATGGTTGCCTCCACGCCTCACACACTTCAAGAGCTTCAGGACACGACTCTTGGCTCA 381

**** * * *********** ****** * ***************** ************

CpEil1a_allele1 CTTTTATCAGCTCTGATGCAGCATTGTGACCCTCCTCAGAGA**CGG**TT**CCC**TCTAGAGAAG 480

CpEil1a_allele2 CTTTTATCAGCTCTGATGCAGCATTGTGACCCTCCTCAGAGA**CGG**TT**CCC**TCTAGAGAAG 480

CpEil1b_allele1 CTTTTATCAGCTCTGATGCAGCATTGTGACCCTCCGCAAAGGCGGTT**CCC**TCTAGAGAAG 441

CpEil1b_allele2 CTTTTATCAGCTCTGATGCAGCATTGTGACCCTCCGCAAAGGCGGTT**CCC**TCTAGAGAAG 441

*********************************** ** ** ******************

CpEil1a_allele1 GGTGTTGCTCCACCATGGTGGCCTAATGGAGATGAAGAGTGGTGGGCCCAGCT**A**GGTCTG 540

CpEil1a_allele2 GGTGTTGCTCCACCATGGTGGCCTAATGGAGATGAAGAGTGGTGGGCCCAGCT**G**GGTCTG 540

CpEil1b_allele1 GGTGTTGCTCCACCATGGTGGCCTAATGGAGATGAAGAGTGGTGGCCCCAGTTAGGTCTG 501

CpEil1b_allele2 GGTGTTGCTCCACCATGGTGGCCTAATGGAGATGAAGAGTGGTGGCCCCAGTTAGGTCTG 501

********************************************* ***** * ******

CpEil1a_allele1 CCAAAGGATCAAGGTCCGCCTCCGTATAAGAAA**CCT**CATGATTTGAAGAAGGCCTGGAAA 600

CpEil1a_allele2 CCAAAGGATCAAGGTCCGCCTCCGTATAAGAAA**CCT**CATGATTTGAAGAAGGCCTGGAAA 600

CpEil1b_allele1 CCAAAAGATCAAGGTCCGCCTCCATATAAGAAA**CCT**CATGATTTGAAGAAGGCCTGGAAA 561

CpEil1b_allele2 CCAAAAGATCAAGGTCCGCCTCCATATAAGAAA**CCT**CATGATTTGAAGAAGGCCTGGAAA 561

***** ***************** ************************************

CpEil1a_allele1 GTCAGTGTTTTAACTGCGGTGATTAAGCACATGTCTCCAGACATTGCGAAGATCCGGAAG 660

CpEil1a_allele2 GTCAGTGTTTTAACTGCGGTGATTAAGCACATGTCTCCAGACATTGCGAAGATCCGGAAG 660

CpEil1b_allele1 GTTAGTGTTTTAACTGCAGTGATCAAACACATGTCACCAGACATTGCGAAAATCCGGAAG 621

CpEil1b_allele2 GTTAGTGTTTTAACTGCAGTGATCAAACACATGTCACCAGACATTGCGAAAATCCGGAAG 621

** ************** ***** ** ******** ************** *********

CpEil1a_allele1 CTTGTTCGGCAGTCGAAATGCTTGCAAGATAAGATGACGGCCAAGGAAAGTGCTACTTGG 720

CpEil1a_allele2 CTTGTTCGGCAGTCGAAATGCTTGCAAGATAAGATGACGGCCAAGGAAAGTGCTACTTGG 720

CpEil1b_allele1 CTCGTTCGGCAGTCGAAATGCTTGCAAGATAAGATGACAGCCAAAGAAAGTGCTACTTGG 681

CpEil1b_allele2 CTCGTTCGGCAGTCGAAATGCTTGCAAGATAAGATGACAGCCAAAGAAAGTGCTACTTGG 681

** *********************************** ***** ***************

CpEil1a_allele1 CTTGCTATCATTAACCAGGAGGAAGCCCTGTCACGAAAACTCTATCCTGACAG**CTGTCCA** 780

CpEil1a_allele2 CTTGCTATCATTAACCAGGAGGAAGCCCTGTCACGAAAACTCTATCCTGACAG**CTGTCCA** 780

CpEil1b_allele1 CTTGCTATTATTAACCAGGAGGAGGCTCTTTCACGAAAGCTCTATCCAGACAGATGTCCA 741

CpEil1b_allele2 CTTGCTATTATTAACCAGGAGGAGGCTCTTTCACGAAAGCTCTATCCAGACAGATGTCCA 741

******** ************** ** ** ******** ******** ***** ******

CpEil1a_allele1 **CCCATGTCCATGG**CTGGTGGAAGTGGGTCCTATCTCATCAGCGATACTAGTGACTATGAT 840

CpEil1a_allele2 **CCCATGTCCATGG**CTGGTGGAAGTGGGTCCTATCTCATCAGCGATACTAGTGACTATGAT 840

CpEil1b_allele1 CCTATGTCCATGGCTGGTGGATGTGGGTCCTATCTCATCAGTGATACTAGCGACTATGAT 801

CpEil1b_allele2 CCTATGTCCATGGCTGGTGGATGTGGGTCCTATCTCATCAGTGATACTAGCGACTATGAT 801

** ****************** ******************* ******** *********

CpEil1a_allele1 GTTGAAGGAATGGAAGATGACCGGGATATTGAACTA**G**AGGAATTCAAGCCACCGCGTGAT 900

CpEil1a_allele2 GTTGAAGGAATGGAAGATGACCGGGATATTGAACTA**C**AGGAATTCAAGCCACCGCGTGAT 900

CpEil1b_allele1 GTAGAAGGTGTGGATGATGACCGGAATATCGAAGTAGAGGAATGCAAGC---CTTGTGAT 858

CpEil1b_allele2 GTAGAAGGTGTGGATGATGACCGGAATATCGAAGTAGAGGAATGCAAGC---CTTGTGAT 858

** ***** **** ********* **** *** ** ****** ***** * *****

CpEil1a_allele1 GTCAATCTTTACAGCTTAGGTATCGGGGGTGCTATTGATAGGCTTGTGATGCAACCACCA 960

CpEil1a_allele2 GTCAATCTTTACAGCTTAGGTATCGGGGGTGCTATTGATAGGCTTGTGATGCAACCACCA 960

CpEil1b_allele1 GTCAATCTTTACAGCTTGGGGATTGGGG**A**TGCTATTGACAGGCTTG**TGATGCAACCACCA** 918

CpEil1b_allele2 GTCAATCTTTACAGCTTGGGGATTGGGG**G**TGCTATTGACAGGCTTG**TGATGCAACCACCA** 918

***************** ** ** **** ********* *********************

CpEil1a_allele1 CCATTGGCCCAAGTAAAGGGGGAGCTTCTTGAAACCAACTCCTCAGATTTTATCCAGAAG 1020

CpEil1a_allele2 CCATTGGCCCAAGTAAAGGGGGAGCTTCTTGAAACCAACTCCTCAGATTTTATCCAGAAG 1020

CpEil1b_allele1 **CCATTG**------------------------------------------------------ 924

CpEil1b_allele2 **CCATTG**------------------------------------------------------ 924

******

CpEil1a_allele1 AGAAAGCAACCATCTGAAAAGCAGCAGCAGGAGCACATGATTATGGAAAAGAAGATATAC 1080

CpEil1a_allele2 AGAAAGCAACCATCTGAAAAGCAGCAGCAGGAGCACATGATTATGGAAAAGAAGATATAC 1080

CpEil1b_allele1 ------------------------------------------------------------ 924

CpEil1b_allele2 ------------------------------------------------------------ 924

CpEil1a_allele1 ACATGCGAGTATACCCAGTGTCCGTATAACAATGCCCGTTTAGGCTTTCAAGACAGATCT 1140

CpEil1a_allele2 ACATGCGAGTATACCCAGTGTCCGTATAACAATGCCCGTTTAGGCTTTCAAGACAGATCT 1140

CpEil1b_allele1 ------------------------------------------------------------ 924

CpEil1b_allele2 ------------------------------------------------------------ 924

CpEil1a_allele1 GCAAGGAACAATCACCAGATGAATTGTCTGTACAGAAGTTGTTCTTCCCAGGGACTTGGT 1200

CpEil1a_allele2 GCAAGGAACAATCACCAGATGAATTGTCTGTACAGAAGTTGTTCTTCCCAGGGACTTGGT 1200

CpEil1b_allele1 ------------------------------------------------------------ 924

CpEil1b_allele2 ------------------------------------------------------------ 924

CpEil1a_allele1 GGCATGTCAAACTTCCAACTTAACAATGAGAAGTCAGCTATCTTCCCCTCTATGCCTTTT 1260

CpEil1a_allele2 GGCATGTCAAACTTCCAACTTAACAATGAGAAGTCAGCTATCTTCCCCTCTATGCCTTTT 1260

CpEil1b_allele1 ------------------------------------------------------------ 924

CpEil1b_allele2 ------------------------------------------------------------ 924

CpEil1a_allele1 GGTCAAACCAAGCTTCTTAATGGTCCTCAATTAGGGAACCAGGCTCCTCCTCCATTCAGT 1320

CpEil1a_allele2 GGTCAAACCAAGCTTCTTAATGGTCCTCAATTAGGGAACCAGGCTCCTCCTCCATTCAGT 1320

CpEil1b_allele1 ------------------------------------------------------------ 924

CpEil1b_allele2 ------------------------------------------------------------ 924

CpEil1a_allele1 GTTTCAAGACTTGGACTACCAGAAGATGGACAGAGAACGATTTCAGATCTAATGTCATTC 1380

CpEil1a_allele2 GTTTCAAGACTTGGACTACCAGAAGATGGACAGAGAACGATTTCAGATCTAATGTCATTC 1380

CpEil1b_allele1 ------------------------------------------------------------ 924

CpEil1b_allele2 ------------------------------------------------------------ 924

CpEil1a_allele1 TACGATTCAAATGTTCAACAGAACCAATGTGGCATCAATTCCGGGAATCTTGATGTTCAT 1440

CpEil1a_allele2 TACGATTCAAATGTTCAACAGAACCAATGTGGCATCAATTCCGGGAATCTTGATGTTCAT 1440

CpEil1b_allele1 ------------------------------------------------------------ 924

CpEil1b_allele2 ------------------------------------------------------------ 924

CpEil1a_allele1 CTAGGAAATCATAATCTGCCACAGCAGCTGCCACAGCAGCAGCCACAGCAGCAGCCACAG 1500

CpEil1a_allele2 CTAGGAAATCATAATCTGCCACAGCAGCTGCCACAGCAGCAGCCACAGCAGCAGCCACAG 1500

CpEil1b_allele1 ------------------------------------------------------------ 924

CpEil1b_allele2 ------------------------------------------------------------ 924

CpEil1a_allele1 CCACAGCCACAGCCACAGACACAGCCACAGACACAGACACAGACACTGACACAGACACAG 1560

CpEil1a_allele2 CCACAGCCACAGCCACAGACACAGCCACAGACACAGACACAGACACAGACACAGACACAG 1560

CpEil1b_allele1 ------------------------------------------------------------ 924

CpEil1b_allele2 ------------------------------------------------------------ 924

CpEil1a_allele1 ACACAGACACAGACACAGACACGGCCACAGCTGCAGCACGAGTTTCAGATTCAACAGCTG 1620

CpEil1a_allele2 ACACAGACACAGACACAGACACGGCCACAGCTGCAGCACGAGTTTCAGATTCAACAGCTG 1620

CpEil1b_allele1 ------------------------------------------------------------ 924

CpEil1b_allele2 ------------------------------------------------------------ 924

CpEil1a_allele1 GATGAAGGCTTCTTCGGAGAGAGTGTAGCAATGGGAGGCAACAATGTATGTGCAGAAACT 1680

CpEil1a_allele2 GATGAAGGCTTCTTCGGAGAGAGTGTAGCAATGGGAGGCAACAATGTATGTGCAGAAACT 1680

CpEil1b_allele1 ------------------------------------------------------------ 924

CpEil1b_allele2 ------------------------------------------------------------ 924

CpEil1a_allele1 AATATCCCCATGCCCTTAAATCATTCTGCTGCTTTCCAACCAACAGATTTCCAATTCAAT 1740

CpEil1a_allele2 AATATCCCCATGCCCTTAAATCATTCTGCTGCTTTCCAACCAACAGATTTCCAATTCAAT 1740

CpEil1b_allele1 ------------------------------------------------------------ 924

CpEil1b_allele2 ------------------------------------------------------------ 924

CpEil1a_allele1 CACTGCAAACCATTTGATTCTGTTTTTGATGCCACCCCAAACGACAACAGTGCCCTGGAC 1800

CpEil1a_allele2 CACTGCAAACCATTTGATTCTGTTTTTGATGCCACCCCAAACGACAACAGTGCCCTGGAC 1800

CpEil1b_allele1 ------------------------------------------------------------ 924

CpEil1b_allele2 ------------------------------------------------------------ 924

CpEil1a_allele1 TTCAGATTTGGTTCCCCTTTCAATATAGGAGCAGTTGATCATTTTGTTATGGATCCACAC 1860

CpEil1a_allele2 TTCAGATTTGGTTCCCCTTTCAATATAGGAGCAGTTGATCATTTTGTTATGGATCCACAC 1860

CpEil1b_allele1 ------------------------------------------------------------ 924

CpEil1b_allele2 ------------------------------------------------------------ 924

CpEil1a_allele1 CCAAAGCAGCAGGACGTATCCATGTGGTACCTTTGAAGGGGAGAAAGACCTTCGTTGGCT 1920

CpEil1a_allele2 CCAAAGCAGCAGGACGTATCCATGTGGTACCTTTGAAGGGGAGAAAGACCTTCGTTGGCT 1920

CpEil1b_allele1 ------------------------------------------------------------ 924

CpEil1b_allele2 ------------------------------------------------------------ 924

CpEil1a_allele1 CATCTTTATTTTGGCTCAAGTAGGTAAATCTTCTTCATGTTTAATACAGTAAGTCATACA 1980

CpEil1a_allele2 CATCTTTATTTTGGCTCAAGTAGGTAAATCTTCTTCATGTTTAATACAGTAAGTCATACA 1980

CpEil1b_allele1 ------------------------------------------------------------ 924

CpEil1b_allele2 ------------------------------------------------------------ 924

CpEil1a_allele1 ATATTACATTTAGTGTATTTCGCTGGATGTTACAAACCGGACCTCTATGGATCTCTGGTC 2040

CpEil1a_allele2 ATATTACATTTAGTGTATTTCGCTGGATGTTACAAACCGGACCTCTATGGATCTCTGGTC 2040

CpEil1b_allele1 ------------------------------------------------------------ 924

CpEil1b_allele2 ------------------------------------------------------------ 924

CpEil1a_allele1 TTTAAATTCTGCATTTGTGTTTTGAAAACAAGTTGAAGCGAGGGGGTTGTATATGGGGAA 2100

CpEil1a_allele2 TTTAAATTCTGCATTTGTGTTTTGAAAACAAGTTGAAGCGAGGGGGTTGTATATGGGGAA 2100

CpEil1b_allele1 ------------------------------------------------------------ 924

CpEil1b_allele2 ------------------------------------------------------------ 924

CpEil1a_allele1 AGGCTCAATCAACCCCATAATTTTCACTTGCTTTCAGTCCTGTGTTGTGTTGCAGACATT 2160

CpEil1a_allele2 AGGCTCAATCAACCCCATAATTTTCACTTGCTTTCAGTCCTGTGTTGTGTTGCAGACATT 2160

CpEil1b_allele1 ------------------------------------------------------------ 924

CpEil1b_allele2 ------------------------------------------------------------ 924

CpEil1a_allele1 TGATAAGTGGTTTGGCTGTGTTATTAGTATGTATACAGATCTTTGTGCTACTTTGGATTG 2220

CpEil1a_allele2 TGATAAGTGGTTTGGCTGTGTTATTAGTATGTATACAGATCTTTGTGCTACTTTGGATTG 2220

CpEil1b_allele1 ------------------------------------------------------------ 924

CpEil1b_allele2 ------------------------------------------------------------ 924

CpEil1a_allele1 TTTAAGGTGTTGTCTGTTTATTTGTAAAAAAAAAAAAAAAAA 2262

CpEil1a_allele2 TTTAAGGTGTTGTCTGTTTATTTGTAAAAAAAAAAAAAAAAA 2262

CpEil1b_allele1 ------------------------------------------ 924

CpEil1b_allele2 ------------------------------------------ 924

**Fig. S1** Nucleotide alignment of the *CpEil1a* (allele 1 and allele 2) and *CpEil1b* (allele 1 and allele 2) isolated from *C. portenschlagiana* ‘PKMp11’used in the present study. The target site of the pEIL1a CRISPR/Cas9 vector construct targeting only *CpEil1a* is shown in turquoise blue and PAM sequences are underlined with a black line. The two target sites of the pEil1ab CRISPR/Cas9 vector construct targeting both *CpEil1a* and *CpEil1b* are shown in yellow and PAM sequences are underlined with a black line. Primers used for amplification of fragments containing the target sites for *CpEil1a* and *CpEil1b* are indicated in black and purple, respectively. Moreover, the forward primers are underlined in green and the reverse primers are underlined in red. Nucleotide differences between alleles are shown in white on black background.
