# Supplementary material for: CRISPR/Cas9‐mediated mutation of Eil1 transcription factor genes affects exogenous ethylene tolerance and early flower senescence in Campanula portenschlagiana
Source: Plant Biotechnol J. 2023 Oct 12;22(2):484–96. doi: 10.1111/pbi.14200 (PMC10826993; doi:10.1111/pbi.14200)
Supplement: Supplementary file 2 — Figure S2 Alignment of the first part of the core DNA‐binding domain of Ein3/Eil proteins [file PBI-22-484-s001.docx]

**α1**  **α2 η1 η2 η3 η4 η5 α3**

Rb_Eil3 172 IGPTPHTLQELQDTTLGSLLSALMQHCDPPQRRFPLEKGVPPPWWPTANEEWWPELGLPKDQGPPPYKKPHDLKKAWKVGVLTAVIKHMSP

Ph_Eil2 173 VGPTPHTLQELQDTTLGSLLSALMQHCDPPQRRFPLEKGVPPPWWPTGQEDWWPQLGLQKDQGPPPYKKPHDLKKAWKVGVLTAVIKHMFP

At_EIN3 171 IGPTPHTLQELQDTTLGSLLSALMQHCDPPQRRFPLEKGVPPPWWPNGKEDWWPQLGLPKDQGPAPYKKPHDLKKAWKVGVLTAVIKHMFP

At_ein3-1 171 IGPTPHTLQELQDTTLGSLLSALMQHCDPPQRRFPLEKGVPPPW*----------------------------------------------

At_ein3-3 171 IGPTPHTLQELQDTTLGSLLSALMQHCDPPQRRFPLEKGVPPPWWPNGKEDWWPQLGLPKDQGPAPYKKPHDLK**N**AWKVGVLTAVIKHMFP

At_EIL1 173 VGPTPHTLQELQDTTLGSLLSALMQHCDPPQRRFPLEKGVSPPWWPNGNEEWWPQLGLPNEQGPPPYKKPHDLKKAWKVGVLTAVIKHMSP

Cp_Eil1b 110 VASTPHTLQELQDTTLGSLLSALMQHCDPPQRRFPLEKGVAPPWWPNGDEEWWPQLGLPKDQGPPPYKKPHDLKKAWKVSVLTAVIKHMSP

Cp_mEil1ab4_b1 110 VASTPHTLQELQDTTLGSLLSALMQHCDPPQRRFPRVLLHHGGLMEMKSGGPS*-------------------------------------

Cp_mEil1ab4_b2 110 VASTPHTLQELQDTTLGSLLSALMQHCDPPQRRFPRGCCSTMVA*----------------------------------------------

Cp_Eil1a 123 MASTPHTLQELQDTTLGSLLSALMQHCDPPQRRFPLEKGVAPPWWPNGDEEWWAQLGLPKDQGPPPYKKPHDLKKAWKVSVLTAVIKHMSP

Cp_mEil1a6_a1^1^  123 MASTPHTLQELQDTTLGSLLSALMQHCDPPQDGSL*-------------------------------------------------------

Cp_mEil1ab4_a1^2^ 123 MASTPHTLQELQDTTLGSLLSALMQHCDPPQRR**----L**GVAPPWWPNGDEEWWAQLGLPKDQGPPPYKKPHDLKKAWKVSVLTAVIKHMSP

Cp_mEil1ab4_a2 123 MASTPHTLQELQDTTLGSLLSALMQHCDPPQRRFPLGRVLLHHGGLMEMKSGGPSWVCQRIKVRLRIRNLMI*------------------

Cm_Eil1 122 MGPTPHTLQELQDTTLGSLLSALMQHCDPPQRRFPLEKGVAPPWWPKGDEEWWAQLGLPKDQGPPPYKKPHDLKK----------------

Ps_Eil3 175 LACTPHTLQELQDTTLGSLLSALMQHCDPPQRRFPLEKGISPPWWPNGNEEWWPQLGLPKDQGPPPYKKPHDLKKAWKVSVLTAVIKHMSP

Rb_Eil2 169 VASTPHTLQELQDTTLGSLLSALMQHCDPPQRRFPLEKGVSPPWWPTGNEEWWPQLNLAN-QGPPPYKKPHDLKKAWKVSVLTAVIKHMSP

Dc_Eil4 166 TGSTPHTLHELQDTTLGSLLSALMQHCDPPQRRFPLEKGIPPPWWPVGDEEWWPQLGIPNDQGPPPYKKPHDLKKAWKVSVLTAVIKHMFP

Dc_Eil2 172 MGSTPHTLQEFQDTTLGSLLSALMQHCDPPQRRFPLEKGHPPPWWPVGNEEWWPQLGIPNDQGPPPYKKPHDLKKAWKVSVLTAVIKHMSP

Cm_eil2 124 IGPTPHTLQELQDTTLDLLLSALIVILYRGVSLSRKVFRPRGGPRGRRNGGLNWVCRKNKGRAHRLTRSLMI*------------------

Ps_Eil2 170 AGPTSHTLQELQDTTLGSLLSALMQHCEPPQRRFPLEKGVPPPWWPNGKEEWWSQLGLSSDHSSPPYKKPHDLKKAWKVGVLTAVIKHMSP

Dc_Eil3 174 IRSSAHALQELQDTTLGSLLSALMQHCDPPQRRFPLEKGLPPPWWPMGNEEWWVELGLPNDYGPPPYKKPHDLKKAWKVSVLTAVIKHMSP

: *:*:*:*****. *********:*********** ***** ..*:** :*.: : . **************.********* *

**__________________________________________**

**Proline rich region ___________**

**BC III**

**Fig. S2** Alignment of the first part of the core DNA-binding domain of Ein3/Eil proteins as defined by Song et al (2015). Numbers given at the front of the sequences are the number of the first residues of the aligned sequences of the individual proteins. The stop codon in At_ein3-1 and the amino acids found after the frameshift in Cm_eil2, Cp_mEil1a6_a1^1^, Cp_mEil1ab4_a1^2^, Cp_mEil1ab4_a2, Cp_mEil1ab4_b1, Cp_mEil1ab4_b2 are shown in white on a black background. The amino acid substitution in At_ein3_3 is shown on a green background and the position of the four missing amino acids and one substitution in Cp_eil1a_4a1 is shown in white on a blue background. These amino acids and missing amino acids in Cm_Eil1 sequences were omitted in the conservation analysis. Helical structures are shown in color (alpha-helices (α1 – 3) in grey and short helical turns (η1 – 5) in yellow). The proline-rich region and the Basic amino acid Domain III are underlined. The alignment was produced from partial EIN3/EIL protein sequences from *Arabidopsis thaliana* (At_EIL1, OAP08200.1; At_EIN3, NP_188713.1), *Campanula medium* (Cm_Eil1, ANH10887.1; Cm_eil2, ANH10888.1), *Campanula portenschlagiana* (Cp_Eil1a, OM925995, Cp_Eil1b, OM925997), *Dianthus caryophyllus* (Dc_Eil2, AAV68140.1; Dc_Eil3, AAV68141.1; Dc_Eil4, AAV68142.1), *Petunia x hybrida* (Ph_Eil2, ATE62978.1), *Paeonia suffruticosa* (Ps_Eil2, AFI61908.1; Ps_Eil3, AFI61909.1), *Rosa x borboniana* (Rb_Eil2, QBM92039.1; Rb_Eil3, QBM92040.1.
