# Supplementary material for: CRISPR/Cas9‐mediated mutation of Eil1 transcription factor genes affects exogenous ethylene tolerance and early flower senescence in Campanula portenschlagiana
Source: Plant Biotechnol J. 2023 Oct 12;22(2):484–96. doi: 10.1111/pbi.14200 (PMC10826993; doi:10.1111/pbi.14200)
Supplement: Supplementary file 3 — Figure S3 Flower longevity and endogenous ethylene production of non‐mutated ‘PKMp11’ and mutated mEil1ab4 flowers [file PBI-22-484-s002.pptx]

## Slide 1
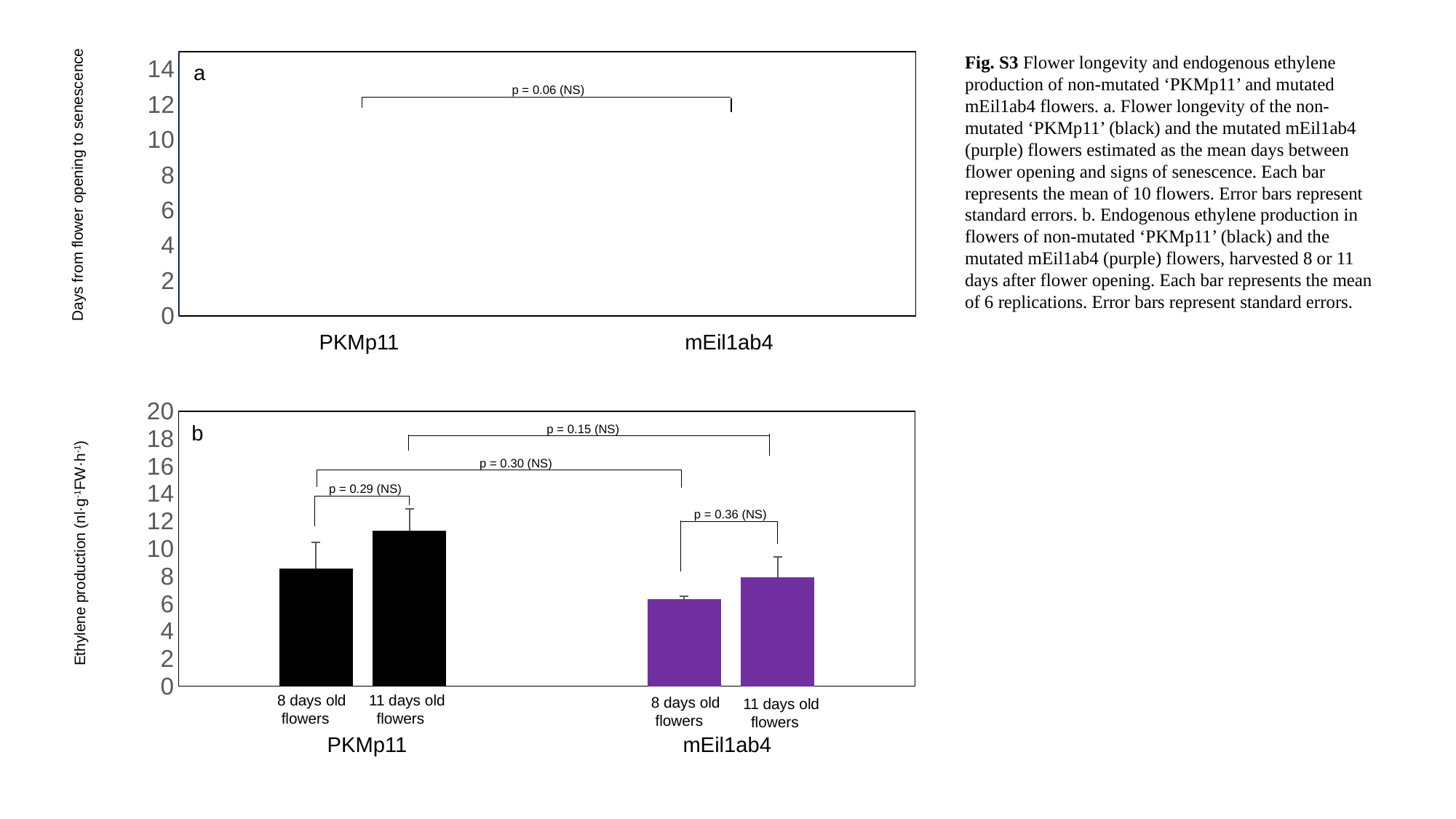

### Chart
| Category | |
|---|---|a
p = 0.06 (NS)
Days from flower opening to senescence
mEil1ab4
PKMp11
### Chart
| Category | | |
|---|---|---|
| Kategori 1 | 8.57 | 11.31 |
| Kategori 2 | 6.31 | 7.9 |b
p = 0.15 (NS)
p = 0.30 (NS)
p = 0.29 (NS)
p = 0.36 (NS)
Ethylene production (nl·g-1FW·h-1)
8 days old
flowers
11 days old
flowers
8 days old
flowers
11 days old
flowers
mEil1ab4
 PKMp11
Fig. S3 Flower longevity and endogenous ethylene production of non-mutated ‘PKMp11’ and mutated mEil1ab4 flowers. a. Flower longevity of the non-mutated ‘PKMp11’ (black) and the mutated mEil1ab4 (purple) flowers estimated as the mean days between flower opening and signs of senescence. Each bar represents the mean of 10 flowers. Error bars represent standard errors. b. Endogenous ethylene production in flowers of non-mutated ‘PKMp11’ (black) and the mutated mEil1ab4 (purple) flowers, harvested 8 or 11 days after flower opening. Each bar represents the mean of 6 replications. Error bars represent standard errors.
