# Supplementary material for: CRISPR/Cas9‐mediated mutation of Eil1 transcription factor genes affects exogenous ethylene tolerance and early flower senescence in Campanula portenschlagiana
Source: Plant Biotechnol J. 2023 Oct 12;22(2):484–96. doi: 10.1111/pbi.14200 (PMC10826993; doi:10.1111/pbi.14200)
Supplement: Supplementary file 4 — Figure S4 Detection of mutations by PCR/RE in CpEil1a of the two F1 plants obtained after cross‐pollination of mEil1a6 with the blue clone ‘5628‐21’ [file PBI-22-484-s003.pptx]

## Slide 1
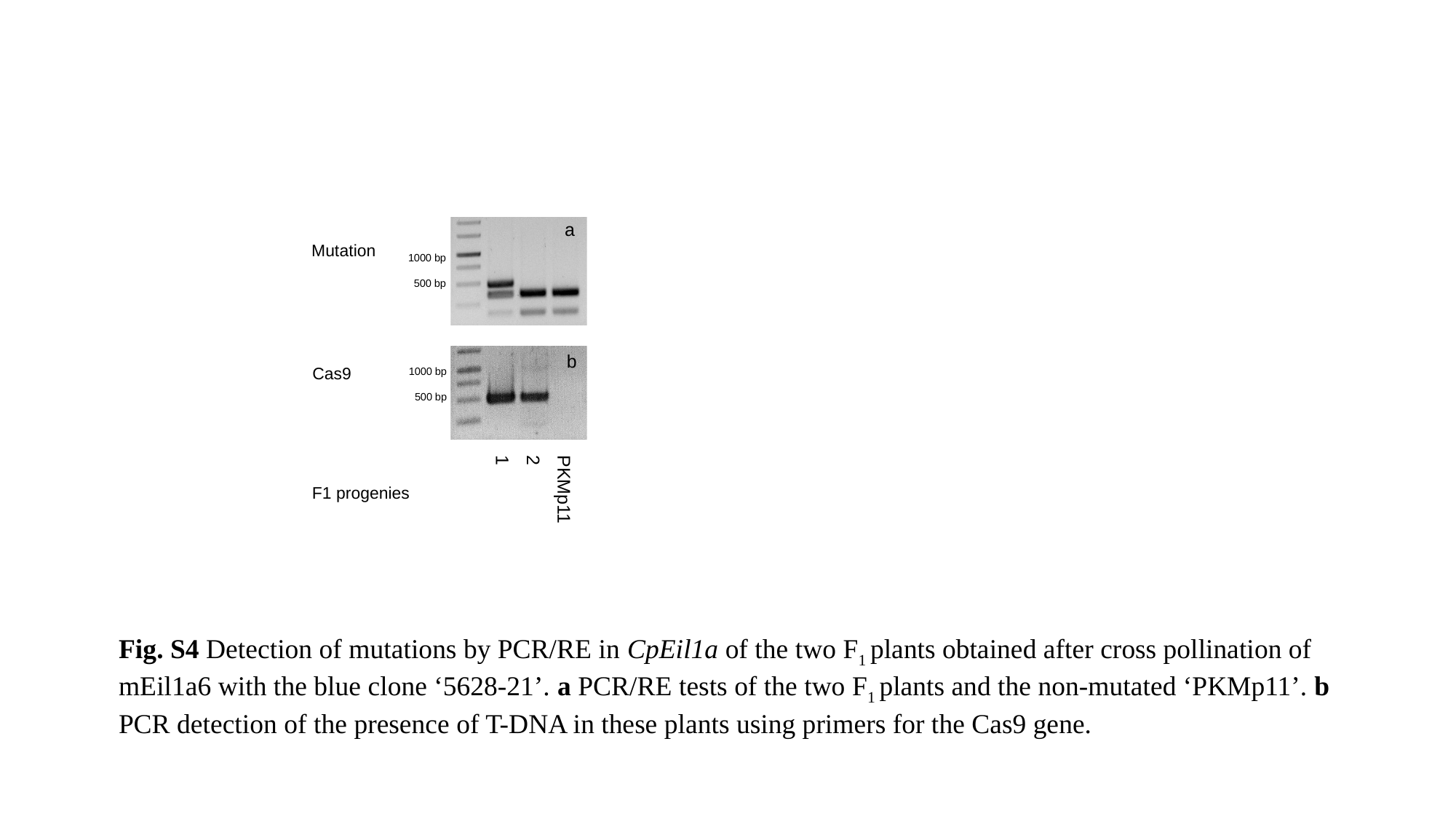

a
Mutation
1000 bp
 500 bp
b
Cas9
1000 bp
 500 bp
PKMp11
2
1
F1 progenies
Fig. S4 Detection of mutations by PCR/RE in CpEil1a of the two F1 plants obtained after cross pollination of mEil1a6 with the blue clone ‘5628-21’. a PCR/RE tests of the two F1 plants and the non-mutated ‘PKMp11’. b PCR detection of the presence of T-DNA in these plants using primers for the Cas9 gene.
