# Supplementary material for: CRISPR/Cas9‐mediated mutation of Eil1 transcription factor genes affects exogenous ethylene tolerance and early flower senescence in Campanula portenschlagiana
Source: Plant Biotechnol J. 2023 Oct 12;22(2):484–96. doi: 10.1111/pbi.14200 (PMC10826993; doi:10.1111/pbi.14200)
Supplement: Supplementary file 5 — Figure S5 Detection of mutations by PCR/RE in CpEil1a and CpEil1b of the progenies from the primary mutant mEil1ab4 (a) of the seven S1 plants obtained after self‐pollination and (b) of the 28 F1 plants obtained after cross‐pollination with the blue clone ‘5628‐21’ [file PBI-22-484-s006.docx]

a

b

**Fig. S5** Detection of mutations by PCR/RE in *CpEil1a* and *CpEil1b* of the progenies from the primary mutant mEil1ab4. **a** PCR/RE tests of seven S_1_ plants obtained after self-pollination of mEil1ab4. **b** PCR/RE tests of the 28 F_1_ plants obtained after crosses between mEil1ab4 and the blue clone ‘5628-21’. F_1_ plants marked with *asterisks* indicate that the mutations in these plants were confirmed by sequencing.
