# Supplementary material for: CRISPR/Cas9‐mediated mutation of Eil1 transcription factor genes affects exogenous ethylene tolerance and early flower senescence in Campanula portenschlagiana
Source: Plant Biotechnol J. 2023 Oct 12;22(2):484–96. doi: 10.1111/pbi.14200 (PMC10826993; doi:10.1111/pbi.14200)
Supplement: Supplementary file 6 — Figure S6 Detection of mutations by PCR/RE in CpEil1a and CpEil1b of the 45 F2 plants [file PBI-22-484-s005.pptx]

## Slide 1
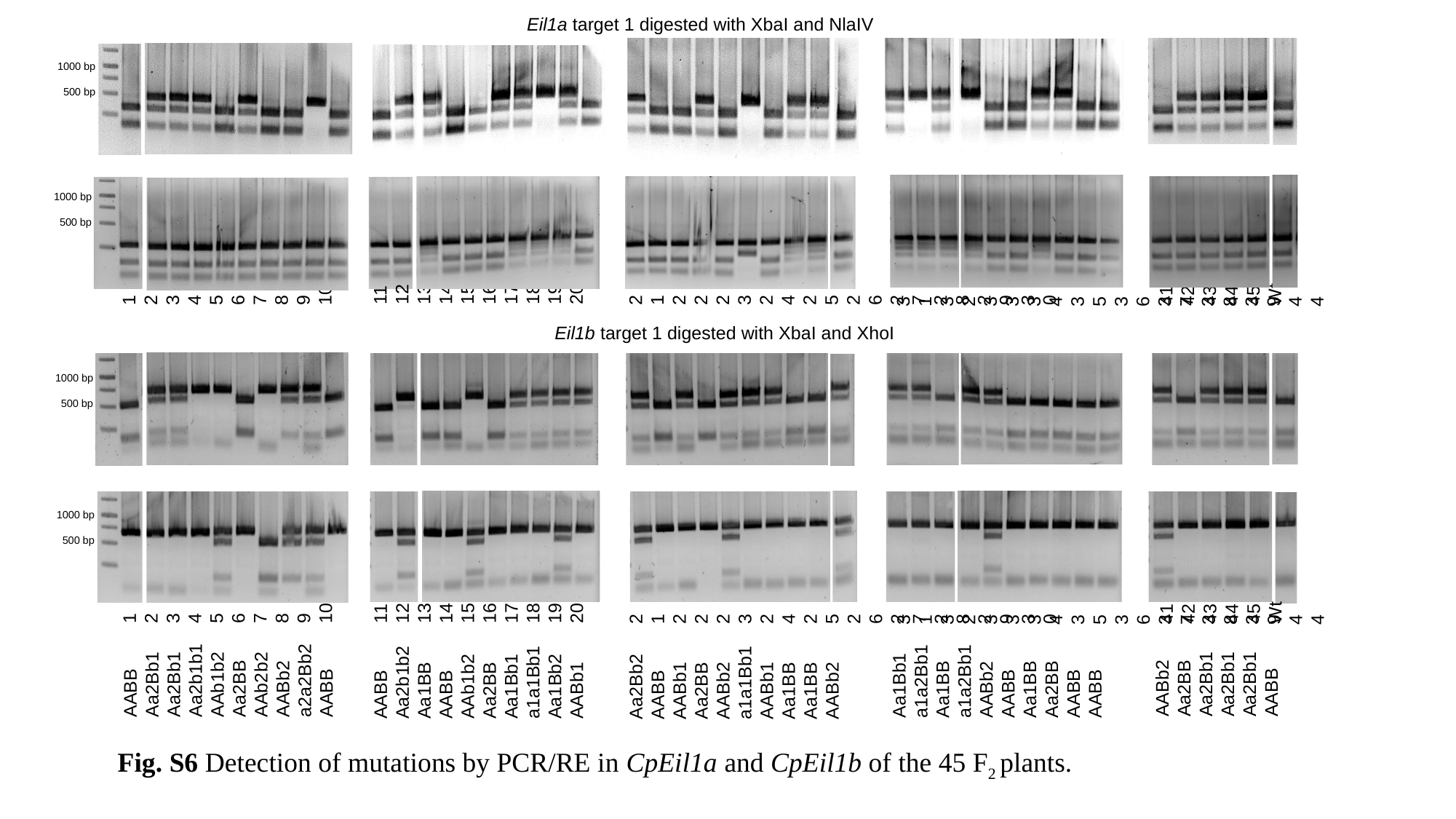

Eil1a target 1 digested with XbaI and NlaIV
1000 bp
 500 bp
1000 bp
 500 bp
41
42
43
44
45
Wt
11
12
13
14
15
16
17
18
19
20
1
2
3
4
5
6
7
8
9
10
21
2223
24
25
26
27
28
29
30
31
32
3334
35
36
37
38
39
44
Eil1b target 1 digested with XbaI and XhoI
1000 bp
 500 bp
1000 bp
 500 bp
41
42
43
44
45
Wt
11
12
13
14
15
16
17
18
19
20
1
2
3
4
5
6
7
8
9
10
21
2223
24
25
26
27
28
29
30
31
32
3334
35
36
37
38
39
44
AABB
Aa2Bb1
Aa2Bb1
Aa2b1b1
AAb1b2
Aa2BB
AAb2b2
AABb2
a2a2Bb2
AABB
Aa1Bb1
a1a2Bb1
Aa1BB
a1a2Bb1
AABb2
AABB
Aa1BB
Aa2BB
AABB
AABB
AABB
Aa2b1b2
Aa1BB
AABB
AAb1b2
Aa2BB
Aa1Bb1
a1a1Bb1
Aa1Bb2
AABb1
Aa2Bb2
AABB
AABb1
Aa2BB
AABb2
a1a1Bb1
AABb1
Aa1BB
Aa1BB
AABb2
AABb2
Aa2BB
Aa2Bb1
Aa2Bb1
Aa2Bb1
AABB
Fig. S6 Detection of mutations by PCR/RE in CpEil1a and CpEil1b of the 45 F2 plants.
