# Supplementary material for: CRISPR/Cas9‐mediated mutation of Eil1 transcription factor genes affects exogenous ethylene tolerance and early flower senescence in Campanula portenschlagiana
Source: Plant Biotechnol J. 2023 Oct 12;22(2):484–96. doi: 10.1111/pbi.14200 (PMC10826993; doi:10.1111/pbi.14200)
Supplement: Supplementary file 7 — Table S1 Fragments created by PCR/RE in the primary mutant mEil1ab4 and progenies [file PBI-22-484-s008.docx]

**Table S1.** Fragments created by PCR/RE in the primary mutant mEil1ab4 and progenies

| Gene | Wild type PCR product  (bp) | Target site | RE | Wild type  AA  (bp) | Wild type (A) and  mutations (a1^2^/a2) | Biallelic for  a1^2^a2  (bp) | Heterozygous  Aa1^2^  (bp) | Heterozygous  Aa2  (bp) | Homozygous  a1^2^a1^2^  (bp) | Homozygous a2a2  (bp) |
| --- | --- | --- | --- | --- | --- | --- | --- | --- | --- | --- |
| Eil1a | 463 | 1 | XbaI | 320,143 | A  a1^2^ (-12)  a2 (-1) | -  451  462 | 320, 143  451  - | 320,143  -  462 | -  451  - | -  -  462 |
| Eil1a | 463 | 1 | NlaIV | 267,136,60 | A  a1^2^ (-12)  a2 (-1) | -  267, 184  267, 136, 59 | 267, 136, 60  267, 184  - | 267, 136, 60  -  267, 136, 59 | -  267, 184  - | -  -  267, 136, 59 |
| Eil1a | 463 | 2 | BspH1 | 215,248 | All WT, no mutation | - | - | - | - | - |
| Gene | Wild type PCR product  (bp) | Target site | RE | Wild type  BB  (bp) | Wild type (B) and  mutations (b1/ b2) | Biallelic for  b1b2  (bp) | Heterozygous  Bb1  (bp) | Heterozygous  Bb2  (bp) | Homozygous  b1b1  (bp) | Homozygous b2b2  (bp) |
| Eil1b | 635 | 1 | XbaI | 492,143 | B  b1 (-7)  b2 (-6+1) | -  628  630 | 492, 143  628  - | 492, 143  -  630 | -  628  - | -  -  630 |
| Eil1b | 635 | 1 | XhoI | 635 | B  b1 (-7)  b2 (-6+1) | -  628  486, 144 | 635  628  - | 635  -  486, 144 | -  628  - | -  -  486, 144 |
| Eil1b | 635 | 2 | BspH1 | 413, 222 | No mutations | - | - | - | - | - |
