# Supplementary material for: CRISPR/Cas9‐mediated mutation of Eil1 transcription factor genes affects exogenous ethylene tolerance and early flower senescence in Campanula portenschlagiana
Source: Plant Biotechnol J. 2023 Oct 12;22(2):484–96. doi: 10.1111/pbi.14200 (PMC10826993; doi:10.1111/pbi.14200)
Supplement: Supplementary file 8 — Table S2 The different combinations of mutated and non‐mutated alleles identified in F2 plants and S1 plants [file PBI-22-484-s004.docx]

**Table S2.** The different combinations of mutated and non-mutated alleles identified in F_2_ plants (progenies after self-pollination of F1 plants) and S_1_ plants (progenies after self-pollination of the mEil1ab4 primary mutant) and the corresponding number of plants tested at each ethylene concentration.

| Generation | No. of mut alleles | Genotypes | No. of plants identified | No. of plants tested** | | |
| --- | --- | --- | --- | --- | --- | --- |
|  |  |  |  | 0.25 ppm | 1.0 ppm | 5.0 ppm |
| F2 | 0 | AABB | 8 | 6 | 5 | 4 |
|  |  | **Total** | **8** | **6** | **5** | **4** |
|  | 1 | AABb2 | 5 | 3 | 4 | 4 |
|  |  | AABb1 | 3 | 3 | 2 | 3 |
|  |  | Aa1^2^BB | 5 | 5 | 4 | 4 |
|  |  | Aa2BB | 5 | 6 | 4 | 4 |
|  |  | **Total** | **18** | **17** | **14** | **15** |
|  | 2 | AAb1b2 * | 2 | 1 | 2 | 2 |
|  |  | AAb2b2 | 1 | 1 | 1 | 1 |
|  |  | Aa1^2^Bb1 | 2 | - | 2 | 2 |
|  |  | Aa1^2^Bb2 | 1 | 1 | 2 | 2 |
|  |  | Aa2Bb1 | 5 | 4 | 4 | 6 |
|  |  | Aa2Bb2 | 1 | - | 1 | - |
|  |  | **Total** | **12** | **7** | **12** | **13** |
|  | 3 | Aa2b1b1 | 1 | 1 | - | 1 |
|  |  | Aa2b1b2* | 1 | - | 2 | 1 |
|  |  | a1^2^a1^2^Bb1 | 1 | 1 | 1 | 1 |
|  |  | a1^2^a2Bb1* | 3 | 1 | 2 | 3 |
|  |  | a2a2Bb2 | 1 | 1 | 1 | 1 |
|  |  | **Total** | **7** | **4** | **6** | **7** |
| S1 | 4 | a1^2^a1^2^b1b2 | 1 | - | 1 | 1 |
|  |  | a1^2^a2b1b1 | 2 | 1 | 2 | 2 |
|  |  | a1^2^a2b1b2 | 1 | - | 1 | 1 |
|  |  | a1^2^a2b2b2 | 1 | 2 | 1 | 1 |
|  |  | a2a2b1b1 | 1 | 1 | - | - |
|  |  | a2a2b1b2 | 1 | 1 | 1 | **-** |
|  |  | **Total** | **7** | **5** | **6** | **5** |
| PKMp11 | 0 | AABB | 28 | 8 | 10 | 10 |
|  |  | **Total** | **28** | **8** | **10** | **10** |

* Outcrossings between F_1_ groups

** Each plant was tested for ethylene tolerance one to five times with five to fifteen days

in between so that new flowers were developed between each test.
