# Supplementary material for: CRISPR/Cas9‐mediated mutation of Eil1 transcription factor genes affects exogenous ethylene tolerance and early flower senescence in Campanula portenschlagiana
Source: Plant Biotechnol J. 2023 Oct 12;22(2):484–96. doi: 10.1111/pbi.14200 (PMC10826993; doi:10.1111/pbi.14200)
Supplement: Supplementary file 9 — Table S3 PCR primers used in this study [file PBI-22-484-s010.docx]

**Table S3** PCR-primers used for cloning of Eil1 partial fragments, for amplification of PCR products containing the CRISPR/Cas9 target sites and for T-DNA detection. All primers are 5´- 3´

|  |  | Size |
| --- | --- | --- |
| anchored 3RACE_rev | GATGATGATGATGGTCGACT_d(T)18 |  |
|  |  |  |
| Cp_EIL1a_fw | GAGCTAGAGAGGAGGATGTGGAGAG | 2262 |
| Tracer_3RACE_rev | GATGATGATGATGGTCGACT |  |
|  |  |  |
| Cp_EIL1b_fw | CTGAGGCGGTTAAAAGAACAAAAC | 924 |
| Cp_EILb_rev | CAATGGTGGTGGTTGCATCACAAGCCTG |  |
|  |  |  |
| tgEil1aF | CATTCAGCCCCTGGGAAGAG | 462 |
| tgEil1aR | CCATGGACATGGGTGGACAG |  |
|  |  |  |
| tgEil1bF | ACCATTCAGTCCCCGGTG | 628 |
| tgEil1bR | CAATGGTGGTGGTTGCATCA |  |
|  |  |  |
| Cas9F | CCAACCACGTCTTCAAAGCA | 467 |
| Cas9R | TCGAGCCTGTGGAAGAATGA |  |
