# Supplementary material for: CRISPR/Cas9‐mediated mutation of Eil1 transcription factor genes affects exogenous ethylene tolerance and early flower senescence in Campanula portenschlagiana
Source: Plant Biotechnol J. 2023 Oct 12;22(2):484–96. doi: 10.1111/pbi.14200 (PMC10826993; doi:10.1111/pbi.14200)
Supplement: Supplementary file 10 — Table S4 Composition of media used for Agrobacterium tumefaciens‐mediated transformation of C. portenschlagiana ‘PKMp11’ [file PBI-22-484-s009.docx]

Table S4. Composition of media used for *Agrobacterium tumefaciens*-mediated transformation

of petioles from *C. portenschlagiana* ‘PKMp11’

| **1 liter** | **Co-culture**  **medium** | **Selection**  **medium** | **Rooting**  **medium** |
| --- | --- | --- | --- |
| MS reduced salts  (Duchefa M0233, no 1B) | 2.3 g | 2.3 g | - |
| Gamborg B5 (Macro, micro, vit)  (Duchefa G0210) | - | - | 3.2 g |
| 2,4-D | 2.0 mg | 2.0 mg | - |
| Thidiazuron | 1.0 mg | 1.0 mg | - |
| CuSO_4_ | - | 2.0 mg | - |
| CoCl_2_ x 8 H_2_O | 2.0 mg | 2.0 mg | - |
| MES | 0.5 g | 0.5 g | - |
| Sucrose | 30 g | 30 g | 30 g |
| Timentin | - | 300.0 mg | 300.0 mg |
| Kanamycin | - | 100.0 mg | 50.0 mg |
| Gelzan^TM^ | 3.5 g | 3.5 g | 3.5 g |
| pH | 6.0 | 6.0 | 6.0 |
